# Disclosing the Biocide Activity of α-Ag_2−2*x*_Cu*_x_*WO_4_ (0 ≤ *x* ≤ 0.16) Solid Solutions

**DOI:** 10.3390/ijms231810589

**Published:** 2022-09-13

**Authors:** Paula Fabiana dos Santos Pereira, Camila Cristina De Foggi, Amanda Fernandes Gouveia, Ivo Mateus Pinatti, Luís Antônio Cabral, Eva Guillamon, Iván Sorribes, Miguel A. San-Miguel, Carlos Eduardo Vergani, Alexandre Zirpoli Simões, Edison Z. da Silva, Laécio Santos Cavalcante, Rosa Llusar, Elson Longo, Juan Andrés

**Affiliations:** 1 CDMF, LIEC, Department of Chemistry, Federal University of São Carlos (UFSCar), P.O. Box 676, São Carlos 13565-905, SP, Brazil; 2 Department of Physical and Analytical Chemistry, University Jaume I (UJI), 12071 Castelló, Spain; 3 Department of Conservative Dentistry, Faculty of Dental Sciences, Federal University of Rio Grande do Sul, Rio Grande do Sul 90035-004, RS, Brazil; 4 Institute of Chemistry, State University of Campinas (Unicamp), Campinas 13083-859, SP, Brazil; 5 Department of Chemistry, Federal University of Maranhao, Avenida dos Portugueses, 1966, São Luís 65080-805, MA, Brazil; 6 Institute of Physics, “Gleb Wataghin” (IFGW), State University of Campinas, Campinas 13083-859, SP, Brazil; 7 Departamento de Materiais Odontológicos e Prótese, Faculdade de Odontologia de Araraquara, São Paulo State University (UNESP), P.O. Box 1680, Araraquara 14801-903, SP, Brazil; 8 Faculty of Engineering of Guaratinguetá, São Paulo State University (UNESP), Guaratinguetá 12516-410, SP, Brazil; 9 PPGQ-GERATEC, Universidade Estadual do Piauí, Rua: João Cabral, N. 2231, P.O. Box 381, Teresina 64002-150, PI, Brazil

**Keywords:** α-Ag_2−2*x*_Cu*_x_*WO_4_ solid solutions, morphology, biocide activity, DFT study

## Abstract

In this work, α-Ag_2−2*x*_Cu*_x_*WO_4_ (0 ≤ *x* ≤ 0.16) solid solutions with enhanced antibacterial (against methicillin-resistant *Staphylococcus aureus*) and antifungal (against *Candida albicans*) activities are reported. A plethora of techniques (X-ray diffraction with Rietveld refinements, inductively coupled plasma atomic emission spectrometry, micro-Raman spectroscopy, attenuated total reflectance–Fourier transform infrared spectroscopy, field emission scanning electron microscopy, ultraviolet–visible spectroscopy, photoluminescence emissions, and X-ray photoelectron spectroscopy) were employed to characterize the as-synthetized samples and determine the local coordination geometry of Cu^2+^ cations at the orthorhombic lattice. To find a correlation between morphology and biocide activity, the experimental results were sustained by first-principles calculations at the density functional theory level to decipher the cluster coordinations and electronic properties of the exposed surfaces. Based on the analysis of the under-coordinated Ag and Cu clusters at the (010) and (101) exposed surfaces, we propose a mechanism to explain the biocide activity of these solid solutions.

## 1. Introduction

Transition metal tungstates belong to an important family of inorganic functional materials with potential applications in various fields [1] and have been widely studied [2,3,4] due to their unique structures and photoluminescence emissions at room temperature [5]. Among them, alpha silver tungstate (α-Ag_2_WO_4_) has received special attention from the scientific community because of its interesting properties, including photoluminescence emission [5,6,7,8,9,10], antimicrobial activity [11,12,13,14,15,16,17,18], and photocatalytic properties [11,13,19,20,21,22,23,24,25]. As a result, α-Ag_2_WO_4_ has found exciting applications in cationic dye removal [16], catalytic systems for the oxidation of cyclohexene to adipic acid [22], electrocatalysis [26,27,28,29], the chemical fixation of CO_2_ gas [30], and as a lubricant [31] and gas sensor [32,33].

The formation of solid solutions, i.e., multicomponent materials by the mixture of two or more crystalline solids, is a widely used strategy to fine-tune colligative properties, crystal structures, band gap values, and optical properties, and has many positive impacts on material functionalities [34], in addition to representing opportunities to understand structure–property relationships [35]. Solid solutions involving the addition of copper (Cu^2+^) in different materials have been reported in the literature; for instance, in TiO_2_ and Ag_3_PO_4_ to enhance photocatalytic activity [36,37], as well as magnetic, electrical, and optical properties in ZnO and MoO_3_ [38,39], and to enhance the optical and magnetic properties of Ca_0.9_Cu_0.01_WO_4_ [40], Zn_1__−*x*_Cu*_x_*WO_4_ [41], Mn_1__−*x*_Cu*_x_*WO_4_ [42], Fe_62__−*x*_Co_10_Y_8_Cu*_x_*B_20_ [43], and DyCo_5__−*x*_Cu*_x_* [44]. Asokan et al. [45] pointed out that substituting Co^2+^, Ni^2+^, and Cu^2+^ cations into YMnO_3_ increased its electric conductivity and magnetism. Din et al. [46] studied the composition of La_0.7_Pr_0.3_Fe_11.4__−*x*_Cu*_x_*Si_1.6_ and verified that a small amount of Cu substitution is capable of tuning performance for application in magnetic refrigerators without loss of hysteresis. Mary et al. [47] investigated the structure and superconducting properties of Cu in NdBa_2_Cu_3__−*x*_M*_x_*O_7+δ_ (M = Fe, Co) and NdBa_2_Cu_3__−*x*_M*_x_*O_7−δ_ (M = Ni, Zn) systems, and found that the phase transition and superconducting properties depend on the type and concentration of Cu^2+^ cations.

α-Ag_2_WO_4_ is a *p*-type semiconductor that displays unique structural and electronic flexibility that allows the substitution of Ag and/or W by different metal cations. In recent years, our research group has been engaged in research to investigate the doping process at the α-Ag_2_WO_4_ [48,49,50,51,52]. In particular, we have investigated α-Ag_2__−2*x*_Zn*_x_*WO_4_ (0 ≤ *x* ≤ 0.25) [49] and α-Ag_2__−2*x*_Ni*_x_*WO_4_ (0 ≤ *x* ≤ 0.08) [48] solid solutions. Very recently, Nobre et al. investigated structure, morphology, optical properties, and photocatalytic performance in the degradation of RhB under blue-light-emitting-device irradiation of Ag_1.98_Cu_0.02_WO_4_ [53]. Ayappan et al. investigated the photocatalytic removal of methylene blue and tetracycline hydrochloride antibiotics using α-Ag_2–2*x*_Cu*_x_*WO_4_ (0 ≤ *x* ≤ 0.12) solid solutions [54]. However, there has been no investigation of a copper-doped α-Ag_2_WO_4_ semiconductor as a biocide material reported in the literature until now.

Motivated by the experimental and theoretical works showing the enhanced photocatalytic activity and to investigate the structure, morphology, and biocide activity related to the substitution of α-Ag_2_WO_4_ microcrystals by Cu^2+^ cations, we report in this study the solid solution synthesis of α-Ag_2*x*_Cu*_x_*WO_4_ (0 ≤ *x* ≤ 0.16) by the co-precipitation (CP) method. These microcrystals were structurally characterized by X-ray diffraction (XRD) with Rietveld refinements, inductively coupled plasma atomic emission spectrometry (ICP-AES), micro-Raman spectroscopy (MR), attenuated total reflectance–Fourier transform infrared spectroscopy (ATR-FTIR), field emission scanning electron microscopy (FE-SEM), ultraviolet–visible spectroscopy (UV–vis), and photoluminescence (PL) emissions. X-ray photoelectron spectroscopy (XPS) was employed to determine the local coordination of Cu^2+^ cations. To fully unlock the potential of these materials, a deeper understanding of their electronic structures is required. In the present work, the experimental results are sustained by first-principles calculations at the density functional theory (DFT) level to decipher the geometry, electronic properties, and magnetism corresponding to the (010) and (101) surfaces of α-Ag_2−2*x*_Cu*_x_*WO_4_ (0 ≤ *x* ≤ 0.16) microcrystals. The performances of the as-synthesized α-Ag_2−2*x*_Cu*_x_*WO_4_ (0 ≤ *x* ≤ 0.16) solid solutions as antibacterial and antifungal agents against methicillin-resistant *Staphylococcus aureus* (MRSA) and *Candida albicans* (*C. albicans*), respectively, were investigated in detail.

From the analysis of the results presented here, we can rationalize how the morphology was modified through the incorporation of Cu^2+^ cations, which were associated with changes in the electronic structures of the exposed surfaces. Furthermore, a relationship between morphology and biocide activity was established to explain cell death.

## 2. Results and Discussion

### 2.1. Structural Analysis of the Cu Atom in the α-Ag_2_WO_4_ Structure

#### 2.1.1. XRD and Rietveld Rietveld Refinement

**Figure 1:**
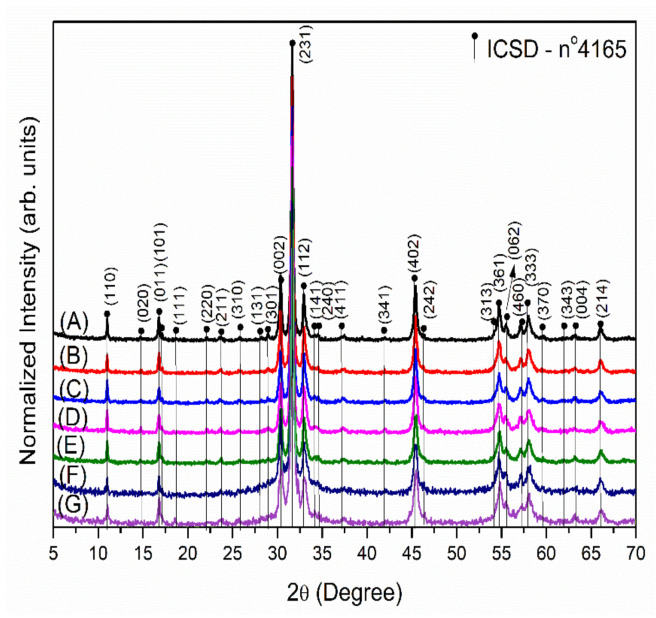
XRD patterns of the α-Ag_2−2*x*_Cu*_x_*WO_4_ solid solutions with *x* = (**A**) 0.00, (**B**) 0.005, (**C**) 0.01, (**D**) 0.02, (**E**) 0.04, (**F**) 0.08, and (**G**) 0.16.

The XRD patterns of the α-Ag_2−2*x*_Cu*_x_*WO_4_ (0 ≤ *x* ≤ 0.16) solid solutions are displayed in Figure 1. In Figure 1A–G, from 2θ = 30.3 to 33.1, a slight shift to higher 2θ values can be observed. This behavior confirmed the successful substitution of the Ag^+^ by the Cu^2+^ cations at the A-site of the orthorhombic α-Ag_2_WO_4_ structure. The results show that all peaks matched those of α-Ag_2_WO_4_ crystals with an orthorhombic structure and the (Pn2n) space group (Inorganic Crystal Structure Database (ICSD), no. 4165). The strong peak associated with the (231) plane suggested that the α-Ag_2−2*x*_Cu*_x_*WO_4_ (0 ≤ *x* ≤ 0.16) solid solutions display high crystallinity. The incorporation of Cu cations did not change the lattice structure, and only a structural distortion was observed; the intensity of the peaks decreased as the amount of Cu cations in the crystalline lattice was increased. From a structural point of view, the differences in the ionic radii between the Ag and Cu cations, 1.15 Å and 0.73 Å, respectively, led to the formation of defects (in the bulk and at the surfaces and interfaces), which decreased the long-range structural ordering.

The orthorhombic α-Ag_2_WO_4_ structure is composed of W atoms coordinated to six oxygen atoms to form a distorted octahedral [WO_6_] cluster. The Ag atoms present different local coordinations, namely, Ag1 and Ag2 (coordination seven), Ag3 (coordination six), Ag4 and Ag5 (coordination four), and Ag6 (coordination two), in which all Ag atoms are located in the centers of distorted clusters, i.e., [AgO_y_] (*y* = 2, 4, 6, and 7) [6,55]. The optimized lattice parameters from the DFT calculations for the α-Ag_2_WO_4_ structure were *a* = 10.78 Å, *b* = 12.60 Å, and *c* = 5.72 Å. These values showed good agreement with the experimental values of *a* = 10.880 Å, *b* = 12.027 Å, and *c* = 5.901 Å, the deviations being 0.92%, 4.52%, and 3.05%, respectively.

**Figure 2:**
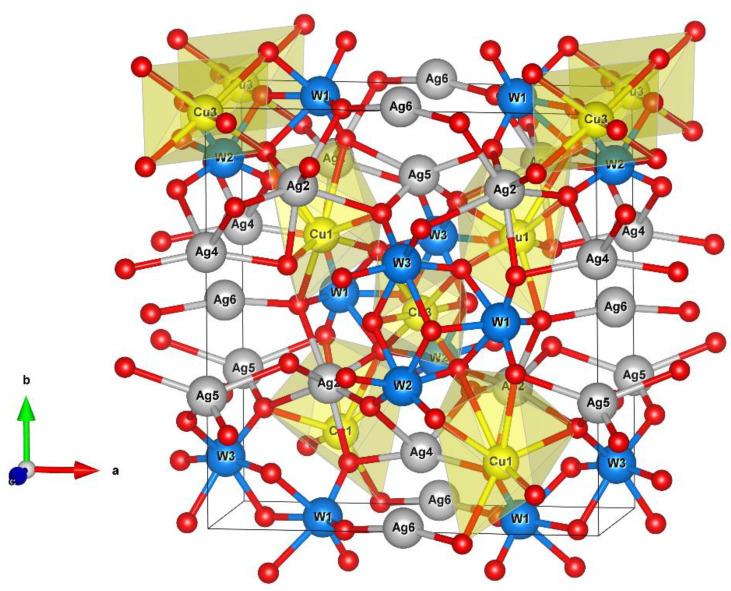
Three-dimensional representation of the orthorhombic α-Ag_2_WO_4_ structure highlighting the [CuO_7_] and [CuO_6_] clusters where the Cu atom substitutions can take place at the Ag sites in the α-Ag_2−2x_Cu*_x_*WO_4_ (0 ≤ *x* ≤ 0.16) structure.

Rietveld refinement was performed to obtain the lattice parameters, unit cell volumes, and atomic positions of the α-Ag_2−2*x*_Cu*_x_*WO_4_ (0 ≤ *x* ≤ 0.16) solid solutions by means of the general structure analysis system software [56] (see Figure S1). The experimental data from the Rietveld refinement are shown in Table S1. An analysis of the results showed a good fit for the values of χ^2^ and R (R_Bragg_, R_wp_, and R_p_). The Rietveld results were consistent with the ICSD N° 4165 pattern, corresponding to an orthorhombic structure, which thus confirmed the XRD results and the absence of additional phases in the α-Ag_2−2*x*_Cu*_x_*WO_4_ (0 ≤ *x* ≤ 0.16) solid solutions. Based on the Rietveld refinement, there were small differences between the unit cell parameters of the samples, which were associated with slight structural distortions. This behavior was due to the process of substitution of Ag by Cu, which triggered a rearrangement in the geometry of the [AgO_y_] by [CuO_y_] clusters and led to the formation of structural defects in the α-Ag_2−2*x*_Cu*_x_*WO_4_ solid solutions. Tables S2–S5 list the values of the atomic coordinates (x, y, z) for Ag, W, O, and Cu atoms obtained by Rietveld refinements. Distortions can be noted in the atomic coordinates (x, y, z) of all atoms. These structural changes are more prominent in the O positions, since they are the lighter atoms and form a 3D framework, which is connected to other cations within the crystalline lattice. On the basis of the Rietveld refinement (see Figure S1 and Tables S2–S5), it was possible to estimate which sites were occupied by Cu^2+^ cations in the α-Ag_2_WO_4_ structure. At lower concentrations, it was not possible to determine the Cu distribution correctly due to the limits of detection and quantification of typical laboratory X-ray diffractometers, which can be 5−10% [57]. Therefore, the Cu site was identified for samples with *x* = 0.08 and 0.16, and, according to these results, for the sample with *x* = 0.08, the substitution of Ag^+^ by Cu^2+^ cations took place in distorted deltahedral [AgO_7_] clusters. In contrast, at *x* = 0.16, the substitution took place in both distorted deltahedral [AgO_7_] and octahedral [AgO_6_] clusters. For each substitution of an Ag^+^ by a Cu^2+^ cation, an Ag vacancy (V_Ag_) was created in the system, which increased the structural and electronic disorder of the samples. Figure 2 depicts a 3D model of the orthorhombic α-AgWO_4_ structure, in which the clusters where the substitution process takes place are displayed.

#### 2.1.2. Theoretical Models

From the Rietveld refinement, several possibilities for the process of substituting the Cu cations in the different positions of the α-Ag_2_WO_4_ unit cell were computationally investigated, and the preferential atomic sites are shown in Figure S2. The processes of substituting Ag by Cu at different sites bring about structural and electronic changes in the crystalline lattice and, consequently, also in its morphological features and optical properties.

#### 2.1.3. XPS Spectroscopy

**Figure 3:**
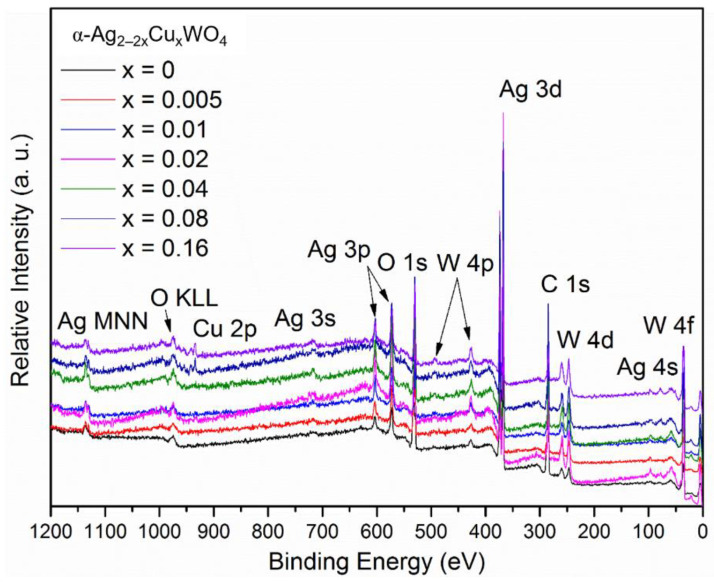
XPS survey spectra of α-Ag_2−2*x*_Cu*_x_*WO_4_ (0 ≤ *x* ≤ 0.16) solid solutions.

XPS spectroscopy was employed to identify the chemical composition, binding energy, and oxidation state of the components present on the surfaces of the materials. The binding energies were obtained by calibrating the spectra using the C-1*s* peak at 284.50 eV. Figure 3 shows the XPS survey spectrum of the α-Ag_2−2*x*_Cu*_x_*WO_4_ (0 ≤ *x* ≤ 0.16) solid solutions. Ag-3*d*, W-4*f*, O-1*s*, and Cu-2*p* peaks were identified for all the samples, and no impurities were detected. The C-1*s* contamination peak can be ascribed to adventitious hydrocarbons from the internal vacuum chamber of the XPS instrument.

Table S6 lists the XPS peak ratios between C, Ag, W, O, and Cu in At% for typical XPS survey spectra of the α-Ag_2−2*x*_Cu*_x_*WO_4_ (0 ≤ *x* ≤ 0.16) solid solutions. The small deviations are due to the degree of insertion of the Cu^2+^ cations into the bulk and surface. In addition, a high-resolution spectrum for each element was obtained to further analyze the surface. The high-resolution Ag-3*d* XPS spectrum in the range of 364–380 eV presented two peaks (see Figure S3a–g). Moreover, this XPS spectrum was deconvoluted into two components located at 367.4 and 373.4 eV (Δ = 6 eV), corresponding to Ag-3*d*_5/2_ and Ag-3*d*_3/2_, thus indicating the presence of Ag^+^ cations. The additional deconvoluted components at 368.2 and 374.2 eV (Δ = 6 eV) were due to the presence of Ag^0^, which can be related to the Ag nanoparticles coated on the surfaces of the materials.

The high-resolution W-4*f* XPS spectra in the range from 32 to 40 eV presented two XPS peaks, as shown in Figure S3h–n. The two components located at 34.8 and 37.0 eV (Δ = 2.2 eV) were attributed to W-4f_7/2_ and W 4f_5/2_, which confirmed the W^6+^ oxidation state. The two components at 34.2 (W-4f_7/2_) and 36.0 eV (W-4f_5/2_) (Δ = 2.2 eV) can be ascribed to W^5+^, and the single component at 40 eV can be assigned to W-5*p*_3/2_. The high-resolution O 1s spectra in the range from 526 to 537 eV are presented in Figure S3o–u. The O-1*s* peaks tended to be broad, with multiple overlapping components, and it was not possible to uniquely fit these components. In general, the peaks located at 529.6, 531.2, and 532.8 eV corresponded to the Ag–O and W–O bonds and the presence of the OH group of the H_2_O molecule, respectively. The broad overlapping peak in the region of ~530 eV was associated with the oxygen in the crystal lattice of the α-Ag_2−2*x*_Cu*_x_*WO_4_ solid solutions.

The high-resolution Cu-2*p* XPS spectra in the range of 926–965 eV is shown in Figure S4a–f. The two component peaks at 934 and 954 eV (Δ = 20 eV) were assigned to Cu-2*p*_3/2_ and Cu-2*p*_1/2_, respectively. Chemical state differentiation using only XPS spectra can be difficult, but the two Cu^2+^ satellite peaks located at 942 and 962 eV (Δ = 20 eV) could be considered signatures of the 2+ oxidation state of the Cu element in the samples. The satellite peaks were associated with a lack of symmetry in the *d*^9^ electronic configuration related to the Jahn–Teller effect.

Moreover, the Cu-2*p*_3/2_ peak in the Cu (II) was shifted and much broader compared to that in the Cu (I). Since copper was found to exist in the Cu^2+^ state, it is reasonable to assume that these Cu^2+^ cations would modify the lattice with concomitant structural distortions along the bonds of the O−W−O−Cu, O−Cu−O frameworks.

#### 2.1.4. ICP-AES Spectroscopy

ICP-AES analysis was used not only to quantify the Cu cations but also to verify the purity of the compounds. The corresponding data are presented in Table 1.

An analysis of the ICP-AES results showed that the theoretical and calculated concentrations (expressed in ppm) were very similar, with only small deviations being detected. Therefore, we can conclude that practically all the added Cu cations were incorporated into the α-Ag2WO4 lattice and that no impurities were observed, in agreement with the XPS spectroscopy analysis.

#### 2.1.5. MR-Raman and ATR-FTIR Spectroscopy

MR-Raman and ATR–FTIR spectroscopies provide information on the degree of structural order/disorder effects at short range. The experimental and theoretical values of the Raman-active modes are presented in Figure S5.

As shown in Figure S5A, there are 19 vibrational Raman-active modes. According to Lin et al. [25], the Raman-active modes in the range from 500 to 1000 cm^−1^ are associated with vibrations in the distorted octahedral [WO_6_] clusters, and those between 100 and 500 cm^−1^ can be assigned to the external vibrational modes of the [AgO_y_] (y = 2, 4, 6, and 7) clusters [25]. The most intense mode at 879.6 cm^−1^ originates from the symmetric stretching vibration of the W–O bonds in the distorted octahedral [WO_6_] clusters [25,58]. Sreedevi et al. [59] reported that the other Raman modes at wavenumbers between that of the most intense mode and 500 cm^−1^ are related to bending modes of the O–W–O moiety as well as inter-chain deformations and lattice modes.

The Raman-active modes associated with an orthorhombic structure confirmed that all the α-Ag_2−2*x*_Cu*_x_*WO_4_ (0 ≤ *x* ≤ 0.16) solid solutions exhibited short-range structural order. However, as revealed by the FWHM, the Raman peak position, and the Raman peak intensity of the α-Ag_2−2*x*_Cu*_x_*WO_4_ (0 ≤ *x* ≤ 0.16) solid solutions, the peaks exhibited broader vibrational modes with the increase in Cu (see Table S7). The line widths of the most intense mode at 879.6 cm^−1^ (A_1g_) Raman-active modes were obtained through deconvolution of the Lorentzian curves.

The line width was observed to show a dependence on the Cu concentration. The α-Ag_2−2*x*_Cu*_x_*WO_4_ (*x* = 0.00, 0.01, and 0.08) solid solutions showed Raman peak widening at 879.4 cm^−1^, and the intensity of this Raman mode decreased with increasing Cu content, which was associated with the process of substituting Ag by Cu (see Figure S5A and Table S7). These substitutions changed the values of the bond lengths in the [AgO_y_] clusters (y = 2, 4, 6, and 7), and the distorted octahedral [WO_6_] clusters. This behavior was confirmed by means of the vibrational Raman-active modes between 100 and 500 cm^−1^, which were associated with the external vibrational modes of the [AgO_y_] clusters (y = 2, 4, 6, and 7). The intensities and shifts of these vibrational Raman-active modes varied with Cu content.

Thus, we concluded that the inclusion of Cu cations in the α-Ag_2_WO_4_ lattice modifies the vibrational modes associated with the stretching, bending, and torsion movements along the framework by the [AgO_y_]–[WO_6_]–[CuO_y_] clusters. The experimental and theoretical values for the Raman-active modes were in good agreement (see Figure S5B). However, small shifts between the Raman modes and the two active modes (A_1g_) of α-Ag_2_WO_4_ were not observed in this work, in contrast to the results reported by Turkovic et al. [60]. These small variations might have occurred due to the effect of phonon confinement, the different synthesis method employed, crystal size, or a doping effect [10,59].

Figure S5C shows the experimental ATR-FTIR spectra of the α-Ag_2−2*x*_Cu*_x_*WO_4_ (0 ≤ *x* ≤ 0.16) solid solutions. ATR-FTIR data were obtained in the range from 400 to 1000 cm^−1^, in which only the characteristic peaks corresponding to the distorted tetrahedral [WO_4_]^2−^ moiety were present. The ATR-FTIR spectra in Figure S5C exhibit intense peaks between 865 and 730 cm^−1^, at 567 cm^−1^, and at 400–450 cm^−1^, which were due to the W–O bonds related to asymmetric stretching vibration modes of the [WO_4_]^2−^ group (see the inset in Figure S5C) [5,10,25]. The vibrational IR-active modes at 681 and 670 cm^−1^ were attributed to the bending modes of the W–O–W moiety, and the band at 631 cm^−1^ was related to the asymmetric stretching of the bridging oxygen atoms in the W_2_O_2_ moiety [48,49]. Moreover, some IR-active modes below 400 cm^−1^ related to active vibrational internal and external modes assigned to the symmetric bending and torsional motion of the distorted [WO_4_]^2−^ group, respectively, as well as to the motion of Cu metal were not observed due to the limit of detection of the equipment.

#### 2.1.6. UV–Vis Spectroscopy and PL Emission

The modifications in the electronic structures of α-Ag_2−2*x*_Cu*_x_*WO_4_ (0 ≤ *x* ≤ 0.16) solid solutions in the medium-range mainly affect the optical properties of materials and can be studied through UV–vis spectroscopy and PL emissions. Therefore, UV–vis absorption can be used to evaluate the formation of intermediate energy levels between the valence band (VB) and the conduction band (CB) caused by defects and/or distortions in the lattice, and, consequently, to determine the band gap energy (*E*_gap_) of the semiconductors. PL measurements provide important information about the structural defect densities in the lattice and can be used to corroborate the UV–vis spectra reported previously in the literature [6,25,61].

**Figure 4:**
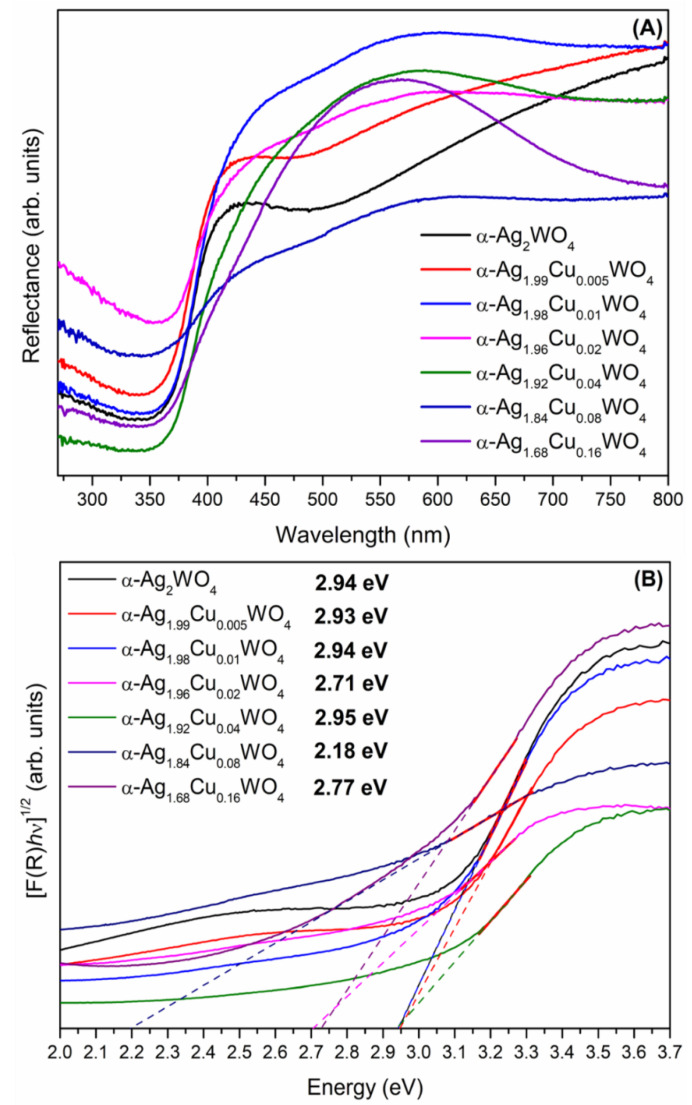
(**A**) UV–vis diffuse reflectance spectra and (**B**) *E*_gap_ values of the α-Ag_2−2*x*_Cu*_x_*WO_4_ (0 ≤ *x* ≤ 0.16) solid solutions.

Figure 4A displays the UV–vis diffuse reflectance and optical *E*_gap_ value spectra of α-Ag_2−2*x*_Cu*_x_*WO_4_ (0 ≤ *x* ≤ 0.16) solid solutions. All samples presented a broad absorption band from 350 to 425 nm. This broad absorption band suggested that the emission process takes place via a multi-level process in the distorted octahedral [WO_6_] clusters [5]. A blue–green shift was observed due to the presence of Cu cations in the α-Ag_2_WO_4_ crystal lattice. According to Sreedevi et al. [5], this exponential optical absorption edge and band gap energy are controlled by the degree of structural disorder in the lattice. Thus, in the α-Ag_2−2*x*_Cu*_x_*WO_4_ (*x* = 0.005 to 0.02) solid solutions, a small blue–green shift in the absorption peak with respect to the pure α-Ag_2_WO_4_ solid solutions was noted, while for the α-Ag_2−2*x*_Cu*_x_*WO_4_ (*x* = 0.04 to 0.16) solid solutions, a small red shift was observed. This behavior can be attributed to the fact that the insertion of small quantities of Cu cations into the α-Ag_2_WO_4_ crystal lattice results in low structural order in the long- and short-range, while for samples doped above *x* = 0.04, greater structural disorder and distortions occur, in agreement with the XRD and Raman data. Therefore, the optical *E*_gap_ values for α-Ag_2−2*x*_Cu*_x_*WO_4_ (0 ≤ *x* ≤ 0.16) solid solutions were obtained in accordance with our previous work [49]. Figure 4B shows the optical *E*_gap_ values, which were obtained by extrapolation of the linear part of the curve according to the Kubelka–Munk [62] equations. Assuming that the α-Ag_2−2*x*_Cu*_x_*WO_4_ (0 ≤ *x* ≤ 0.16) solid solutions underwent indirect allowed electronic transitions, the *E*_gap_ values for the samples were calculated using *n* = 2. The experimental *E*_gap_ values displayed an important decrease, as was observed for all of the α-Ag_2−2*x*_*M_x_*WO_4_ (*M* = Cu, Ni, and Zn) systems [48,49], thus demonstrating that the *M*-cations in the α-Ag_2_WO_4_ crystal lattice promote distortions in the short, medium, and long ranges, and consequently generated defects in the lattice that influenced the optical properties of the material. Moreover, the substitution process involving Cu cations inside the α-Ag_2_WO_4_ crystal lattice influenced its *E*_gap_ values more strongly than the incorporation of Ni and Zn cations [48,49].

Moreover, this behavior was probably due to the electric and magnetic properties of Cu, as well as Jahn–Teller distortion. This substitution of Ag by Cu ions was accompanied by a reduction in the *E*_gap_ energy due to the formation of intermediate energy levels in the forbidden band, with a concomitant increase in the structural disorder. This effect resulted in a symmetry-breaking process due to the changes in bond lengths and bond angles throughout the framework composed of the [AgO_y_]–[WO_6_]–[CuO_y_] clusters, which, in turn, created intermediate levels in the band gap [5,10,25,50]. The calculated band gap value for pristine α-Ag_2_WO_4_ crystals was 2.92 eV, while the experimental band gap obtained was 2.94 eV (0.7% deviation), showing excellent agreement between the theoretical calculations and experimental measurements.

**Figure 5:**
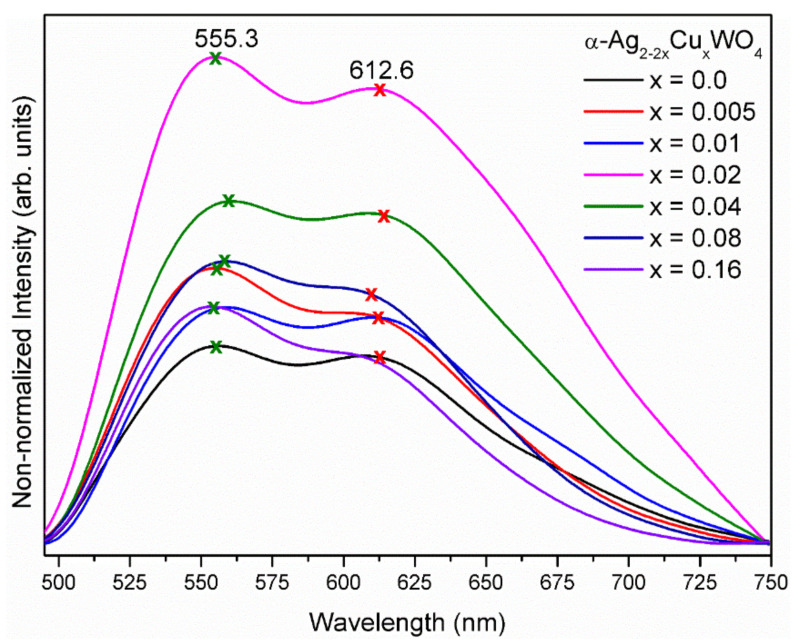
PL emissions of the α-Ag_2−2*x*_Cu*_x_*WO_4_ (0 ≤ *x* ≤ 0.16) solid solutions, excited at 405 nm with a krypton ion laser.

Figure 5 shows the PL emissions of the α-Ag_2−2*x*_Cu*_x_*WO_4_ (0 ≤ *x* ≤ 0.16) solid solutions under 405 nm excitation in the emission range from 490 to 750 nm. Broad band profiles were observed with maximum emissions in the green range and orange–red region, at around 555.3 and 612.6 nm, respectively.

The PL emission broad bands are typical of a multilevel emission process and occurred due to the presence of intermediate energy levels inside the forbidden band region as a result of the distortion of [AgO_y_]–[WO_6_]–[CuO_y_] clusters and defects at medium range [25,49,59] within the α-Ag_2−2*x*_Cu*_x_*WO_4_ solid solution lattice.

However, in the α-Ag_2−2*x*_Cu*_x_*WO_4_ (0.005 ≤ *x* ≤ 0.16) solid solutions, a green shift was observed, and an increase in the emission intensity was also observed for the samples of α-Ag_2−2*x*_Cu*_x_*WO_4_ (0.005 ≤ *x* ≤ 0.02) solid solutions. Above this concentration, the PL emission intensity decreased. The introduction of Cu cations into the α-Ag_2_WO_4_ lattice caused different degrees of distortion in different [WO_6_] clusters in response to the changes in the bond lengths and bond angles along the chain formed by the [AgO_y_]–[WO_6_]–[CuO_y_] clusters. Consequently, the defect density and non-radiative electron–hole recombination rates increased, as was reflected directly in the reduction in the PL intensity.

The effect of the substitution of Ag by Cu cations inside the α-Ag_2_WO_4_ lattice on the PL properties was studied and confirmed by the deconvolution of the PL spectrum using the Voigt area function. Figure S6 presents the deconvolution of the PL spectra and the areas of the respective curves of the α-Ag_2−2*x*_Cu*_x_*WO_4_ (0 ≤ *x* ≤ 0.16) solid solutions. In these PL spectra, three components at 545 nm (green), 597 nm (yellow), and 653 nm (red) were observed, covering the entire visible electromagnetic spectrum. The samples with increased numbers of Cu ions in the α-Ag_2_WO_4_ lattice showed different behavior; the yellow–orange components were clearly the largest in these samples, while in the α-Ag_1.96_Cu_0.02_WO_4_ sample, the red component was the greatest. Therefore, PL emission can be related to several factors, such as intrinsic defects caused by the order or disorder of the system [63], extrinsic defects associated with oxygen vacancies [10], and changes in the morphology (i.e., different shapes and sizes) of the particles, among others. Several authors have proposed that the emissions at 555.3 and 612.6 nm occur due to the charge transfer from distorted ${\left[WO_{6}\right]}_{d}^{x}$ to undistorted ${\left[WO_{6}\right]}_{o}^{x}$ clusters [25,59].

The structural and electronic order/disorder can be associated with the formation of acceptor and donor levels between the CB and VB that may have been introduced by the presence of oxygen vacancies $\left(V_{o}^{z}\right)$ in the lattice. Using the Kröger–Vink notation [64], the oxygen vacancy can be formed in three different charge states $(V_{o}^{z}=V_{o}^{x}$, $V_{o}^{•}$, and $V_{o}^{••})$; in terms of constituent clusters, there are $\left[WO_{x}\cdot V_{o}^{z}\right]$, $\left[AgO_{x}\cdot V_{o}^{z}\right]$, and $\left[CuO_{x}\cdot V_{o}^{z}\right]$. These oxygen vacancies generate deep- and shallow-level defect states in the forbidden band region with concomitant shifts in the location and intensity of the PL emission. An analysis of Figure S6 suggested the appearance of a synergistic effect between the intrinsic and extrinsic defects in the α-Ag_2−2*x*_Cu*_x_*WO_4_ (0 ≤ *x* ≤ 0.16) solid solutions promoted by the substitution of Ag by Cu cations, with the concomitant appearance of different PL emissions. However, we believe that the deep-level defect states associated with the formation of oxygen vacancies, i.e., $\left[AgO_{x}\cdot V_{o}^{z}\right]$ and $\left[CuO_{x}\cdot V_{o}^{z}\right]$ clusters, are more prominent in an α-Ag_2−2*x*_Cu*_x_*WO_4_ (0 ≤ *x* ≤ 0.16) lattice, favoring the yellow–orange emission and a decrease in the intensity of the PL emission.

### 2.2. Antibacterial and Antifungal Activities

**Figure 6:**
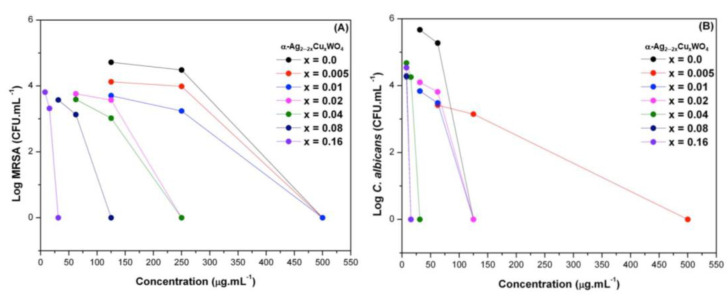
Antibacterial and antifungal activities against (**A**) MRSA and (**B**) *C. albicans* of the α-Ag_2−2*x*_Cu*_x_*WO_4_ (0 ≤ *x* ≤ 0.16) solid solutions.

In this study, we report, for the first time, the antibacterial and antifungal activity of the Cu-substituted α-Ag_2_WO_4_ against two strains of microorganisms: a Gram-positive bacterium and a fungus. Figure 6 shows the corresponding results for the α-Ag_2−2*x*_Cu*_x_*WO_4_ (0 ≤ *x* ≤ 0.16) solid solutions. All samples were effective agents against MRSA and *C. albicans*. As the concentrations of Cu^2+^ cations increased in these materials, the minimum bactericidal and fungicidal concentrations, i.e., the MBCs and MFCs, respectively, decreased.

The pure α-Ag_2_WO_4_ material showed inhibition of MRSA and *C. albicans* at concentrations of 500 and 125 μg/mL, respectively, while the α-Ag_1.68_Cu_0.16_WO_4_ showed inhibition at concentrations of 31.25 and 15.62 μg/mL for MRSA and *C. albicans*, respectively. Then, the solid solutions presented enhanced antimicrobial activity that could be related to the capacity of the material to penetrate the membranes of the microorganisms, as well as its ability to change the microenvironment by releasing reactive oxygen species (ROS), which induce DNA and bacterial membrane damage [65,66,67,68,69].

### 2.3. DFT Calculations

**Figure 7:**
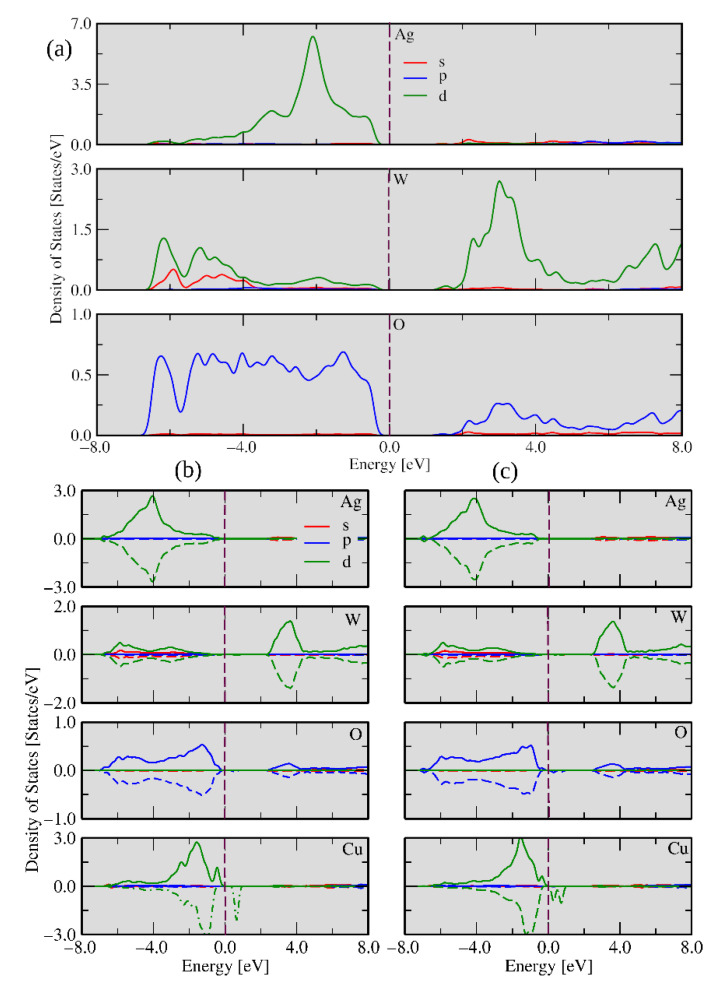
Calculated densities of states for the (**a**) undoped α-Ag_2_WO_4_, (**b**) α-Ag_1.72_Cu_0.14_WO_4_, and (**c**) α-Ag_1.34_Cu_0.33_WO_4_ models.

To obtain a deeper insight into the electronic properties of α-Ag_2−2*x*_Cu*_x_*WO_4_, two theoretical models were constructed: one with the Cu^2+^ cation substitution at the Ag1 site to represent the α-Ag_1.72_Cu_0.14_WO_4_ solid solution, while the substitution at the Ag1 and Ag3 sites rendered the α-Ag_1.34_Cu_0.33_WO_4_ solid solution, in agreement with the results obtained by the Rietveld refinements (see Figure S2). From the theoretical results, the substitution of the Cu^2+^ cation at the Ag1 site was energetically preferred to the substitution at the Ag3 site. The densities of states (DOSs) for these models are shown in Figure 7.

Figure 7a depicts the α-Ag_2_WO_4_ DOS per atom for the pristine system, while the bottom panel shows the DOSs for the Cu-doped case. In Figure 7b,c, the DOSs of α-Ag_1.72_Cu_0.14_WO_4_ and α-Ag_1.34_Cu_0._33WO_4_ are displayed, respectively. The results indicate that the main contributions near the Fermi energy for α-Ag_2_WO_4_ were composed of the *d*-Ag, *d*-W, and *p*-O orbitals, and for the doped models (α-Ag_1.72_Cu_0.14_WO_4_ and α-Ag_1.34_Cu_0.33_WO_4_) there was also the presence of the *d*-Cu orbitals. The valence band maxima (VBMs) for α-Ag_2_WO_4_, Ag_1.72_Cu_0.14_WO_4_, and α-Ag_1.34_Cu_0.33_WO_4_ were formed by *p*-O, *d*-Ag, and *d*-Cu states, while *s*-W, *d*-Cu, and *d*-Cu levels define the conduction band minima (CBMs). The defects associated with the Ag vacancies and the presence of Cu^2+^ cations introduced unoccupied down states near the Fermi energy.

### 2.4. Morphology and Surface Composition: What Really Is Important?

**Figure 8:**
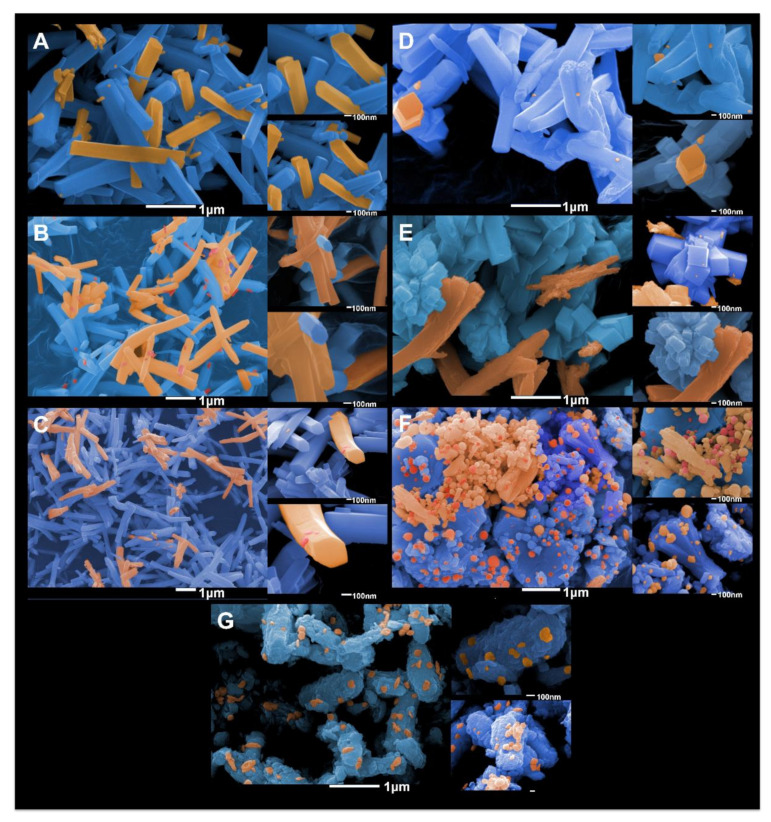
FE-SEM images of α-Ag_2−2*x*_Cu*_x_*WO_4_ microcrystals: (**A**) *x* = 0.00, (**B**) *x* = 0.005, (**C**) *x* = 0.01, (**D**) *x* = 0.02, (**E**) *x* = 0.04, (**F**) *x* = 0.08, and (**G**) *x* = 0.16.

Figure 8A–G display the FE-SEM images. It is known that the α-Ag_2_WO_4_ phase exhibits a long prisms or needles with bases similar to a hexagon [9,70]. This morphology was observed for the α-Ag_2_WO_4_ and α-Ag_2−2*x*_Cu*_x_*WO_4_ (*x* = 0.005, 0.01, and 0.02) microcrystals, which have different sizes, as illustrated in Figure 8A–D. In the α-Ag_2−2*x*_Cu*_x_*WO_4_ (*x* = 0.02) microcrystals, rods with curved surfaces and quasi-square shapes were also detected (see Figure 8D).

Figure 8E–G show FE-SEM images of the α-Ag_2−2*x*_Cu*_x_*WO_4_ (x = 0.04, 0.08, and 0.16) microcrystals. These microcrystals displayed a nanoplate-like morphology and a decrease in particle size which was related to the compound α-Ag_1.92_Cu_0.04_WO_4_.

The FE-SEM images reveal the corresponding surfaces that compose these experimental morphologies. When comparing these crystal shapes with the set of morphologies present in the map of morphologies published by us [71], it was possible to affirm that the long prisms or needles with bases similar to a hexagon is composed of the (010), (001), and (101) surfaces. In contrast, the quasi-square shape is formed only of the (010) and (101) surfaces. This observation supplied the crucial information that the amount of Cu^2+^ cations in the α-Ag_2−2*x*_Cu*_x_*WO_4_ (*x* = 0.04, 0.08, and 0.16) samples increases the stability of the (101) surface for the (001) surface, resulting in the change in its morphology, which can be associated with an increase in the stability of the (101) surface with respect to the (001) surface. Therefore, these solid solutions present (101) and (001) surfaces.

**Figure 9:**
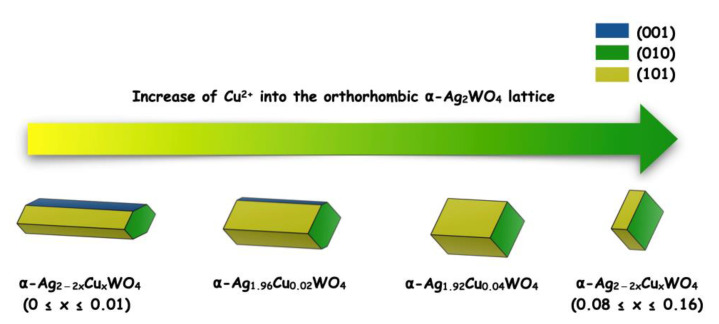
Morphology evolution as a function of the Cu^2+^ content along the α-Ag_2−2*x*_Cu*_x_*WO_4_ solid solution.

From the values for surface energy (*E*_surf_) calculated [71] by means of the Wulff construction [72], we were able to obtain a morphological evolution as a function of Cu^2+^ content, as illustrated in Figure 9. This strategy has been applied successfully in different semiconductors [73,74,75,76]. Analyzing these results and comparing them with the data obtained on the bactericidal properties, we can conclude that: (a) Cu^2+^ modulates the morphology of the solid solution; and that (b) as the contribution of the (001) surface at the morphology increases, the biocide activity also increases.

**Figure 10:**
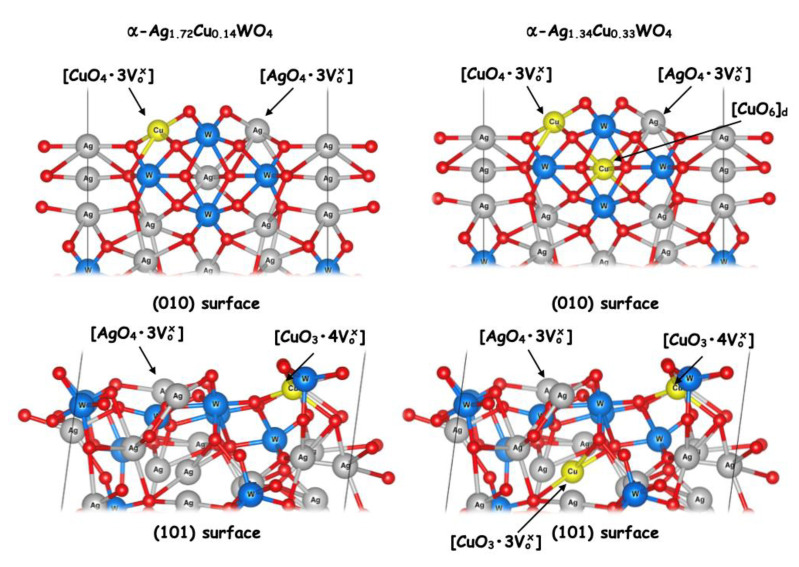
Views of the (010) and (101) surfaces and the Ag1 and Ag6 sites where the substitution process of Ag^+^ by Cu^2+^ cations take place.

As noted previously, the solid solution with a higher amount of Cu^2+^ cations (α-Ag_1.68_Cu_0.16_WO_4_) presented better inhibition of *C. albicans* and MRSA, and these microcrystals exhibit the quasi-square shape that is formed by (010) and (101) surfaces. In what follows, we describe how we computationally examined the effects of Cu doping on the geometry and electronic structure of the exposed (010) and (101) surfaces in this morphology. Figure 10 displays the undercoordinated Ag and Cu clusters present in the (010) and (101) surfaces for both theoretical models (α-Ag_1.84_Cu_0.08_WO_4_ and α-Ag_1.68_Cu_0.16_WO_4_).

In the α-Ag_1.84_Cu_0.08_WO_4_ model, the Cu substitution occurs only in the Ag clusters at the top of each surface, while in the α-Ag_1.68_Cu_0.16_WO_4_ model, the substitution takes place also in the Ag cluster located inside the surface structure.

#### 2.4.1. Kröger–Vink Notation for the Exposed Surfaces

An analysis of Figure 10 revealed that the (010) and (101) surfaces present undercoordinated [AgO_7_] and [AgO_6_] clusters on the top of the surface where the Cu substitution takes place, with simultaneous formation of the corresponding undercoordinated Cu clusters and oxygen vacancies. These vacancies can be described using the Kröger–Vink notation [64]. In this representation, the vacancy corresponds to $V_{a}^{b}$, where *b* is the effective charge and *a* is the occupied crystalline site. Therefore, the atomic clusters with undercoordinated atoms presented in the last layer of the surface can be described as having neutral oxygen vacancies ($V_{o}^{x}$), where the super index x means “neutral” in the Kröger–Vink notation.

For the α-Ag_1.84_Cu_0.08_WO_4_ model, undercoordinated $\left[{\mathrm{AgO}}_{4}\cdot 3V_{o}^{•}\right]$ and $\left[{\mathrm{CuO}}_{4}\cdot 3V_{o}^{•}\right]$ clusters as well as a $\left[{\mathrm{WO}}_{6}\right]$ cluster were observed on top of the (010) surface, while undercoordinated $\left[{\mathrm{AgO}}_{4}\cdot 3V_{o}^{•}\right]$ and $\left[{\mathrm{CuO}}_{3}\cdot 4V_{o}^{•}\right]$ clusters could be found in the (101) surface. In the α-Ag_1.68_Cu_0.16_WO_4_ model, Cu atoms were also present in the Ag6 site. The cluster $\left[{\mathrm{CuO}}_{6}\right]$ was present in the (010) surface, while an undercoordinated $\left[{\mathrm{CuO}}_{3}\cdot 3V_{o}^{•}\right]$ cluster was observed in the (101) surface.

#### 2.4.2. Electronic and Magnetic Structure of the (010) and (101) Surfaces

**Figure 11:**
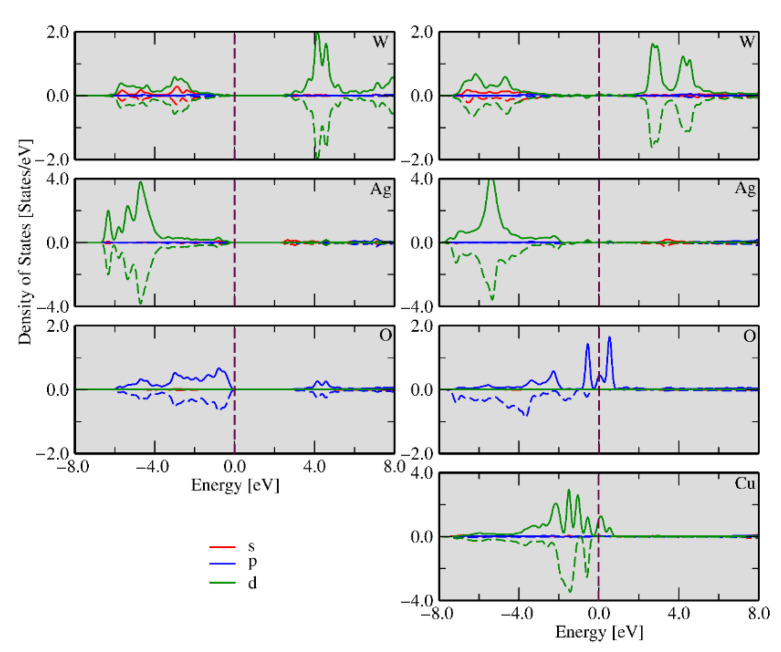
Density of states calculations decomposed in s (red), p (blue), and d (green) states for the (010) surface without (**left**) and with (**right**) Cu impurities.

**Figure 12:**
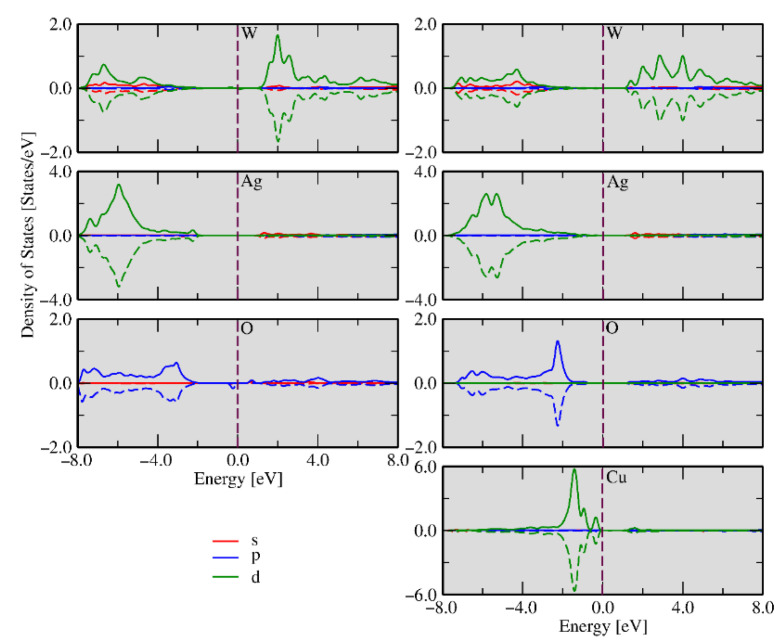
Density functional theory investigation s (red), p (blue), and d (green) states calculated for the (101) surface without (**left** panel) and with (**right** panel) Cu impurities.

An analysis of the previous results shows that oxygen vacancies are common defects in the clusters at the exposed surfaces. The different type of clusters on each surface may have different properties and functions depending on the number and nature of defects. Precise control of electron density at these sites enables surface chemistry regulation. These clusters can be taken as active sites where the biocide activity occurs and determine whether the material will present high or low activity. The different types of clusters on each surface may have different properties and functions depending on the number and nature of defects. The density of states (DOS) was calculated for undoped and Cu-doped Ag_1.72_Cu_0.14_WO_4_ systems in the (010) and (101) surfaces, as shown in Figure 11 and Figure 12, respectively. These calculations are an effective method for studying the changes in electronic properties before and after Cu doping. It can be seen from the left panel of Figure 11 that the *d*-W, s-Ag, and *p*-O orbital states are the major contributions in the conduction band, while the *d*-W, *d*-Ag, and *p*-O orbital states are the significant contributions in the valence band, which can be inferred from the highest electron density concentrated in the 4*d*-Ag orbital.

The calculated band gap for the undoped (010) surface was 2.91 eV. After Cu doping, there is a decrease in the band gap due to the appearance of new levels created by the defects in the forbidden region, which facilitate the electron excitation to these new energy levels (traps). On the other hand, these electrons can move to the surface clusters, facilitating interaction with the environment. An analysis of the right part of Figure 11 showed that the main contributions in the conduction band were the *p*-O, *s*-Ag, and *d*-Cu orbitals, and the shape of its peak can be attributed to the orbital hybridization between *d*-Cu-*p*-O and *d*-Ag-*p*-O. Additionally, the occupied d-W spin-up and spin-down states near the Fermi energy were observed, while *d*-Ag spin-up states in the valence band were noted. The stronger hybridization between the *p*-O and *d*-Cu orbitals produced localized states in the spin-up and spin-down channels, representing a local magnetic moment. We could also observe that the spin charge density was distributed over the entire geometry; however, the more substantial contribution was located at the Cu, O, and Ag atoms of the surface.

The DOS of the (101) surface is displayed in Figure 12, and different aspects could be observed after the Cu doping process. The undoped (in the left) and doped (in the right) systems showed similar densities of states in comparison with the undoped (010) surface. However, occupied spin-down states and spin-up states for *p*-O near the Fermi energy were observed for the undoped case, displaying 1.86 eV of band gap energy. When the impurity was introduced in the (101) geometry, Cu-polarized states appeared near the Fermi energy but doped and undoped (101) surfaces did not present magnetism. In both the (010) and (101) surfaces, the undoped cases presented CBM composed of the *d*-W and *p*-O states, while *d*-Cu and *d*-W were the CBM states for doped surfaces, respectively. These results can explain why α-Ag_1.72_Cu_0.14_WO_4_ presented the most considerable biocide activity. Following the evolution of morphology as a function of Cu content (Figure 9), there is a direct correlation between the presence of (010) surface and biocide activity.

### 2.5. A Proposed Mechanism for the Biocide Activity

ROS are formed as byproducts of the reduction of molecular oxygen (O_2_). They mostly consist of radicals (species with one or more unpaired electrons), such as the superoxide anions (•O_2_^−^) and hydroxyls (•OH) that are generated at the surfaces of metal oxides via different surface-related processes [77], even in the dark [78]. A semiconductor releases an e^−^ from the conduction band and interacts with O_2_ from the environment, generating •O_2_^−^. Simultaneously, an e^−^ moves from the valence band to the conduction band. The decrease in the electron density of the valence band induces the interaction with the H_2_O in the environment. This interaction results from breaking the O−H bond of H_2_O, forming a hydroxyl radical (•OH) and a proton (H^+^) which interacts with •O_2_^−^ to form •O_2_H. These ROS are capable of killing microorganisms by oxidizing and breaking down their cell walls and membranes, and these redox processes take place on the exposed surfaces of the semiconductor [79].

In our case, the undercoordinated Ag and Cu clusters at the top of the (010) and (101) surfaces of the α-Ag_1.68_Cu_0.16_WO_4_ structure (see Figure 10) are the sources of high and low electron density, respectively. They, then, are responsible for the formation of •OH and •O_2_H, which potentially damage membranes and cell walls, leading to cell death. Based on the analysis of these clusters, a mechanism of action is proposed involving the formation of ROS from H_2_O and O_2_ associated with the biocide activity of α-Ag_2−2*x*_Cu*_x_*WO_4_ (0 ≤ *x* ≤ 0.16) solid solutions.

(1) $$\left[{\mathrm{WO}}_{6}\right]+O_{2}\to •O_{2}^{-}$$

(2) $$\left[{\mathrm{AgO}}_{4}\cdot 3V_{o}^{•}\right]+H_{2}O\to H^{+}+•\mathrm{OH}$$

(3) $$\left[{\mathrm{CuO}}_{4}\cdot 3V_{o}^{•}\right]+H_{2}O\to H^{+}+•\mathrm{OH}$$

(4) $$\left[{\mathrm{CuO}}_{3}\cdot 4V_{o}^{•}\right]+H_{2}O\to H^{+}+•\mathrm{OH}$$

(5) $$H^{+}+•O_{2}^{-}\to •O_{2}H$$

These results show a relationship between the morphology of α-Ag_2−2*x*_Cu*_x_*WO_4_ microcrystals and the enhanced biocide activity of α-Ag_1.68_Cu_0.16_WO_4_. The structural and electronic defects increase at the surface; then, the active sites at the exposed surfaces can churn out a plethora of ROS to provoke oxidative stress. The incorporation of Cu on α-Ag_2_WO_4_ contributes to the improvement of antibacterial and antifungal activity in several ways: (i) by reducing the band gap; (ii) by altering the CB position of α-Ag_2_WO_4_; and (iii) by improving the conductivity of α-Ag_2_WO_4_ and enhancing the mobility of charge carriers.

## 3. Methods and Materials

### 3.1. Synthesis of α-Ag_2__−2x_Cu_x_WO_4_ Solid Solutions

α-Ag_2__−2*x*_Cu*_x_*WO_4_ (*x* = 0.00, 0.005, 0.01, 0.02, 0.04, 0.08, and 0.16) solid solutions were prepared by the CP method in accordance with our previous work [49]. Quantities of 2 × 10^−3^ moles of silver nitrate (AgNO_3_; 99.8% purity, Sigma-Aldrich, St. Louis, MI, USA) were dissolved in 50 mL of deionized water at 80 °C under magnetic stirring, and then copper nitrate hydrate (Cu(NO_3_)_2_·*x*H_2_O; 99.99% purity, Sigma-Aldrich) was added in the molar ratios listed above. This solution was added to 50 mL of 1 × 10^−3^ mol of tungstate sodium dihydrate (Na_2_WO_4_·2H_2_O; 99.5% purity, Sigma-Aldrich) previously dissolved at the same temperature, and the mixture was subjected to constant magnetic stirring for 30 min. The α-Ag_2__−2*x*_Cu*_x_*WO_4_ powders thus obtained were washed several times with deionized water and dried in an oven at 70 °C.

### 3.2. Characterizations of α-Ag_2__−2x_Cu_x_WO_4_ Solid Solutions

α-Ag_2__−2*x*_Cu*_x_*WO_4_ (0 ≤ *x* ≤ 0.16) solid solutions were structurally characterized by XRD patterns using a D4 Endeavor, Bruker-AXS with Cu-Kα radiation (λ = 1.5406 Å) in the 2θ range from 10° to 70° in the normal routine, while in the Rietveld routine, a D/Max-2000PC diffractometer Rigaku (Japan) with Cu-Kα radiation (λ = 1.5406 Å) was used in the 2θ range from 10° to 110° with a scanning velocity of 1°/min. XPS was performed using a ScientaOmicron ESCA+ spectrometer with a high-performance hemispheric analyzer (EA 125) with monochromatic Al Kα (hν = 1486.6 eV) radiation as the excitation source. The operating pressure in the ultrahigh vacuum chamber during analysis was 2 × 10^−9^ mbar. Energy steps of 50 and 20 eV were used for the survey and high-resolution spectra, respectively. The ICP-AES analyses were performed using an Agilent^®^ 7500 CX (Santa Clara, CA) with He as the collision gas in the octopolar reaction system to remove all interferences present in the matrix. MR spectroscopy was conducted using a model NRS-3100 (Jasco, Tokyo, Japan) spectrometer with optic microscopy and a refrigerated CCD device detector (−65 °C) operating at λ = 613 nm in the range from 50 to 1000 cm^−1^. ATR-FTIR spectroscopy was recorded in the range from 400 to 1000 cm^−1^ in an FTIR-6200 (Jasco^®^) model spectrophotometer in transmittance mode. The shapes and sizes of the α-Ag_2__−2*x*_Cu*_x_*WO_4_ microcrystals were observed with an FE-SEM Inspect F50 (FEI Company, Hillsboro, OR, USA) operated at 10 kV. The optical properties of the α-Ag_2__−2*x*_Cu*_x_*WO_4_ solid solutions were measured using UV–vis diffuse reflectance spectroscopy and PL measurements at room temperature. UV–vis spectra were obtained using a spectrophotometer (Varian, Palo Alto, CA, USA, model Cary 5G) in diffuse-reflectance mode. PL measurements at room temperature were performed using a Monospec 27 monochromator (Thermal Jarrell Ash, Waltham, MA, USA) coupled to an R446 photomultiplier (Hamamatsu Photonics, Shizuoka, Japan). A krypton-ion laser (Coherent Innova 90K; λ = 350.7 nm) was used as the excitation source, and its maximum output power was maintained at 500 mW.

### 3.3. Antibacterial and Antifungal Activity Measurements

The antibacterial and antifungal activities of the α-Ag_2__−__2*x*_Cu*_x_*WO_4_ (0 ≤ *x* ≤ 0.16) solid solutions were analyzed against two reference strains from the American Type Culture Collection (ATCC): *C. albicans* (ATCC 90028) and MRSA (ATCC 33591). For this purpose, both microorganisms were kept frozen at −80 °C until the time of the experiments. Measurement of the minimal inhibitory concentrations (MICs) and minimal fungicidal/bactericidal concentrations (MFCs/MBCs) for planktonic cells, according to the Clinical and Laboratory Standards Institute broth microdilution method (documents M27-A3 (2008) and M7-A7 (2006)) for yeast and bacteria, respectively [80,81], were performed. The *C. albicans* was thawed and coated on plates containing Sabouraud Dextrose Agar (SDA, HiMedia, Mumbai) culture medium supplemented with chloramphenicol (0.05 g/L) and incubated for 24 h at 37 °C. Then, five colonies were transferred and grown using yeast nitrogen base (YNB) medium supplemented with 100 mM glucose and incubated at 37 °C for 16 h. Subsequently, the cultures were diluted in fresh YNB (1:10 dilution) and incubated at 37 °C until the mid-log growth phase was reached. For MRSA, the cells were thawed and incubated on Mueller–Hinton agar plates for 24 h at 37 °C. Five colonies were then transferred and grown using Mueller–Hinton broth and incubated at 37 °C for 8 h. After this, the cells were diluted in fresh Mueller–Hinton broth (1:10 dilution) and incubated at 37 °C until the mid-log growth phase was reached. The microorganisms were spectrophotometrically standardized to a final concentration of 107 colony-forming units per milliliter (CFU/mL). The MICs were the lowest concentrations of each of the α-Ag_2__−__2*x*_Cu*_x_*WO_4_ (0 ≤ *x* ≤ 0.16) solid solutions that could achieve complete inhibition of growth (no visible growth by visual inspection), and the MFCs/MBCs were defined as the lowest concentrations of each of the α-Ag_2__−__2*x*_Cu*_x_*WO_4_ (0 ≤ *x* ≤ 0.16) solid solutions that resulted in no microbial growth on the plates [12,18,80,81,82]. The MICs and MFCs/MBCs were determined by incubating the microorganisms for 24 h at 37 °C on 96-well microtiter plates exposed to serial dilutions of each of the α-Ag_2__−__2*x*_Cu*_x_*WO_4_ (0 ≤ *x* ≤ 0.16) solid solutions, from 1000 to 0.061 μg/mL, in specific culture media. The MFCs/MBCs were determined by inoculating 25 μL aliquots taken from 10-fold serial dilutions (10^−1^ to 10^−8^) of each well on plates containing SDA for *C. albicans* and Mueller−Hinton agar for MRSA. The plates were incubated at 37 °C for 24 h, and then CFU/mL values were determined. Positive controls were inoculated with the microbial suspension but no α-Ag_2__−__2*x*_Cu*_x_*WO_4_ (0 ≤ *x* ≤ 0.16) solid solutions, while the negative controls consisted of uninoculated culture medium.

### 3.4. Theoretical Approach and Computational Details

DFT calculations were performed [83,84] by employing the semi-local Perdew–Burke–Ernzerhof [85] exchange and correlation energy functional within the spin-polarized generalized gradient approximation (GGA) formulation, as implemented in the Viena ab initio simulation package (VASP) [86,87] version 5.4.4 (VASP Software GmgH, Vienna, Austria). The Kohn–Sham equations were solved using the projector augmented wave (PAW) method [88,89], employing the following projectors: Ag (4*d*^10^, 5*s*^1^), W (5*p*^6^, 5*d*^4^, 6*s*^2^), O (2*s*^2^, 2*p*^4^), and Cu (3*d*^10^, 4*s*^1^), where the valence states are shown in parentheses. The equilibrium volume of α-Ag_2_WO_4_ crystals was obtained by minimizing the stress tensor using a plane wave cutoff of 834 eV, while for the atomic force optimizations, a plane wave cutoff of 469 eV was used. For the Brillouin zone integration, we employed a 1 × 1 × 2 *k*-mesh for stress tensor and atomic force optimizations, while a mesh of twice the size was used for the electronic properties. In all calculations, a Gaussian smearing of 0.01 eV was used, and the atoms could relax until all forces were smaller than 0.01 eV/Å on every atom. To improve the description of the electronic structure, we adopted the GGA + U method proposed by Dudarev et al. [90], in which Hubbard *U* corrections of 5.10 eV and 4.6 eV were used for the *d*-Ag and p-O states, respectively. The relative stability of the models was calculated by comparing the total energies of the Cu^2+^ doped in the α-Ag_2_WO_4_ structures after the optimization calculation. The electronic and structural properties were analyzed for three different theoretical models: a pure α-Ag_2_WO_4_ system and two solid solutions models: α-Ag_1.72_Cu_0.14_WO_4_ and α-Ag_1.34_Cu_0.33_WO_4_, in which one Ag^+^ cation was replaced by one Cu^2+^ cation and, in turn, one Ag vacancy was also created to preserve stoichiometry. In the α-Ag_1.72_Cu_0.14_WO_4_ model, the Cu^2+^ cation replaced the Ag^+^ cation at the Ag1 site, forming a distorted deltahedral [CuO_7_] cluster. In the α-Ag_1.34_Cu_0.33_WO_4_ model, two Cu^2+^ cations were located at the Ag1 and Ag3 sites, forming distorted deltahedral [CuO_7_] and octahedral [CuO_6_] clusters, respectively.

## 4. Conclusions

Solid solutions involving pure α-Ag_2_WO_4_ and α-Ag_2−2*x*_Cu*_x_*WO_4_ compounds present versatile properties resulting from the disorder inherent in their crystal structure, but the fate of biocide activity remains poorly understood. To fully unlock the potential of these materials, in this work we have presented a joint experimental and theoretical study on the structure and electronic properties of α-Ag_2−2*x*_Cu*_x_*WO_4_ (0 ≤ *x* ≤ 0.16) solid solutions, thereby enabling a more accurate and direct comparison between theory and experiment to find a correlation between morphology and biocide (antibacterial, against MRSA, and antifungal, against *C. albicans*) activity.

The main conclusions can be summarized as follows: (i) α-Ag_2−2*x*_Cu*_x_*WO_4_ (0 ≤ *x* ≤ 0.16) solid solutions were synthesized by a low-cost, environmentally friendly chemical co-precipitation method; (ii) their structures (short-, medium-, and long-range) and electronic properties were obtained; (iii) FE-SEM images showed that the morphologies of the samples with low concentrations of Cu atoms were composed of the (010), (001), and (101) surfaces and that the morphologies were hexagonal and rod-like; (iv) an increase in the Cu content provoked a change in the morphology to a nanoplate-like morphology formed only of (010) and (101) surfaces; (v) the increase in Cu in the α-Ag_2_WO_4_ structures enhanced the antibacterial and antifungal activities of α-Ag_2−2*x*_Cu*_x_*WO_4_ (0 ≤ *x* ≤ 0.16) solid solutions against MRSA and *C. albicans*, respectively; (vi) the α-Ag_2−2*x*_Cu*_x_*WO_4_ semiconductor enabled the binding of bacteria and fungi on the exposed surfaces, which resulted in membrane damage due to the presence of ROS, and finally cell death; (vii) the experiments and simulations presented allowed us to propose a plausible mechanism induced by Cu surfaces to explain the biocide activity, based on the presence of undercoordinated Cu clusters in the (010) and (101) surfaces as the active catalytic sites responsible for the formation of ROS; (viii) these results demonstrate that surface-structural engineering of semiconductors via the formation of solid solutions is an effective strategy to enhance biocide efficiencies; and (ix) this work provides new insights into the relationships between the structural, electronic, and magnetic properties of the exposed surfaces at the morphological level, and suggests that electronic and spin selectivity can be used to design other materials as biocide agents.

## Figures and Tables

**Table 1:** ICP-AES data for the α-Ag_2−2*x*_Cu*_x_*WO_4_ (0.005 ≤ *x* ≤ 0.16) solid solutions.

α-Ag_2−*x*_Cu*_x_*WO_4_	Theoretical (ppm)	Experimental (ppm)
*x* = 0.005	0.300	0.354
*x* = 0.01	0.634	0.687
*x* = 0.02	1.118	1.379
*x* = 0.04	2.114	2.778
*x* = 0.08	4.564	5.631
*x* = 0.16	9.860	11.574

## Supplementary Materials

The supporting information can be downloaded at: https://www.mdpi.com/article/10.3390/ijms231810589/s1.
