# Supplementary material for: Disclosing the Biocide Activity of α-Ag2−2xCuxWO4 (0 ≤ x ≤ 0.16) Solid Solutions
Source: Int J Mol Sci. 2022 Sep 13;23(18):10589. doi: 10.3390/ijms231810589 (PMC9504239; doi:10.3390/ijms231810589)
Supplement: Supplementary file 1 [file ijms-23-10589-s001.zip › ijms-1886999-supplementary.pdf]

Disclosing the biocide activity of α-$Ag_{2-2x}Cu_xWO_4$ ($0 \leq x \leq 0.16$) solid solutions

Paula Fabiana dos Santos Pereira[1,2], Camila Cristina De Foggi[3], Amanda Fernandes Gouveia[2,4], Ivo Mateus Pinatti[5], Luís Antônio Cabral[6], Eva Guillamon[2], Iván Sorribes[2], Miguel A. San-Miguel[4], Carlos Eduardo Vergani[7], Alexandre Zirpoli Simões[8], Edison Z. da Silva[6], Laécio Santos Cavalcante[9], Rosa Llusar[2], Elson Longo[1], Juan Andrés[2*]

[1]CDMF, LIEC, Department of Chemistry, Federal University of São Carlos (UFSCar), P.O. Box 676, 13565-905 São Carlos, SP, Brazil
[2]Department of Physical and Analytical Chemistry, University Jaume I (UJI), Castelló 12071, Spain
[3]Federal University of Rio Grande do Sul, Faculty of Dental Sciences, Department of Conservative Dentistry, 90035-004, Rio Grande do Sul, RS, Brazil
[4]Institute of Chemistry, State University of Campinas (Unicamp), 13083-859 Campinas, SP, Brazil
[5]Department of Chemistry, Federal University of Maranhao, Avenida dos Portugueses, 1966, São Luís, MA, 65080-805, Brazil
[6]Institute of Physics, "Gleb Wataghin" (IFGW), State University of Campinas, 13083-859 Campinas, SP, Brazil
[7]São Paulo State University (UNESP), P. O. Box 1680, 14801-903, Araraquara-SP, Brazil
[8]Faculty of Engineering of Guaratinguetá, São Paulo State University (UNESP), 12516-410, Guaratinguetá, SP, Brazil
[9]PPGQ-GERATEC, Universidade Estadual do Piauí, Rua: João Cabral, N. 2231, P.O. Box 381, 64002-150, Teresina–PI, Brazil
*Corresponding author. E-mail address: andres@qfa.uji.es

Supplementary Material (SM)

Figures

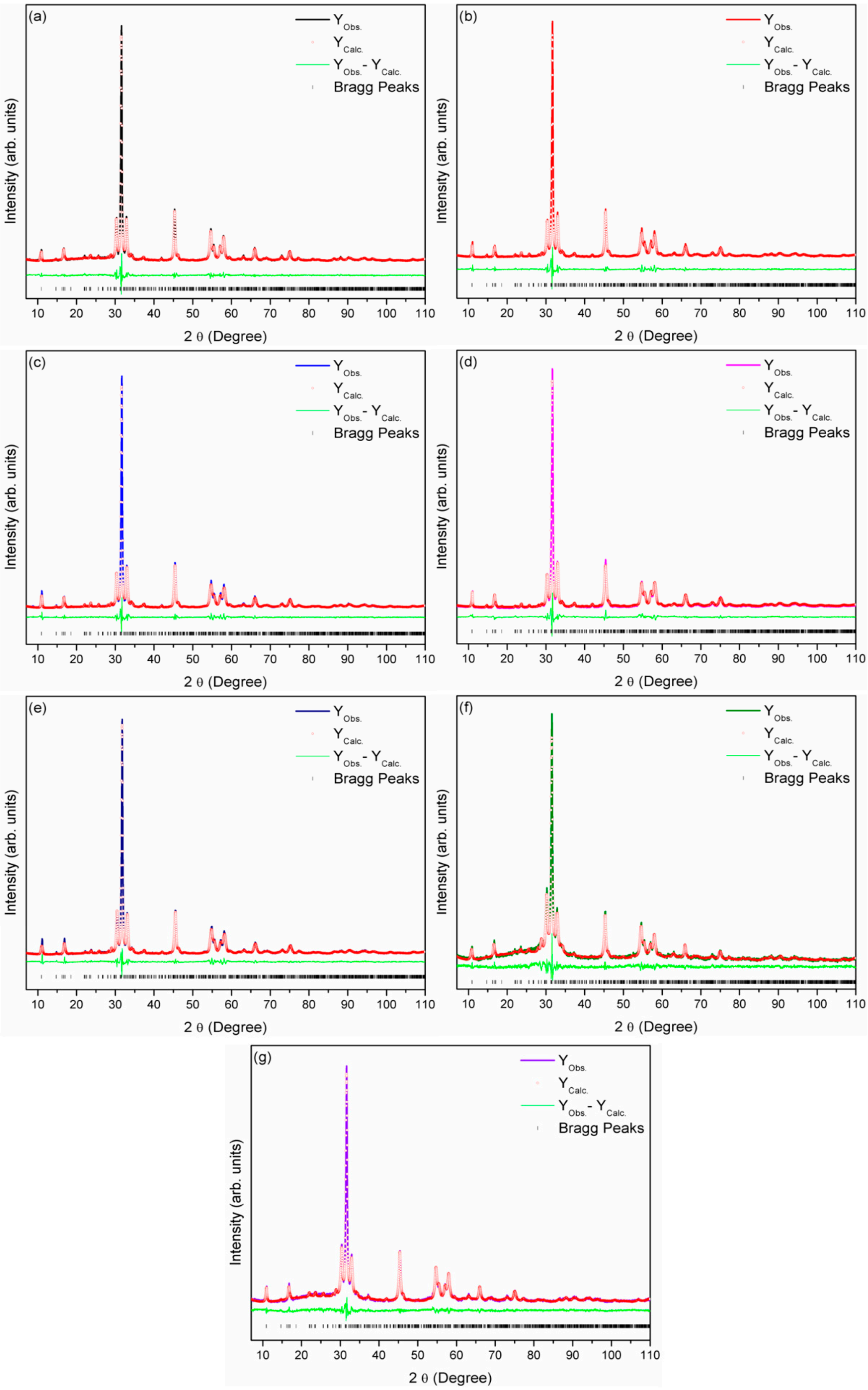

Figure S1. Rietveld refinement plot of the *α*-$Ag_{2-2x}Cu_xO_4$ solid solutions with: (a) $x = 0.00$, (b) $x = 0.005$, (c) $x = 0.01$, (d) $x = 0.02$, (e) $x = 0.04$, (f) $x = 0.08$, and (g) $x = 0.16$.

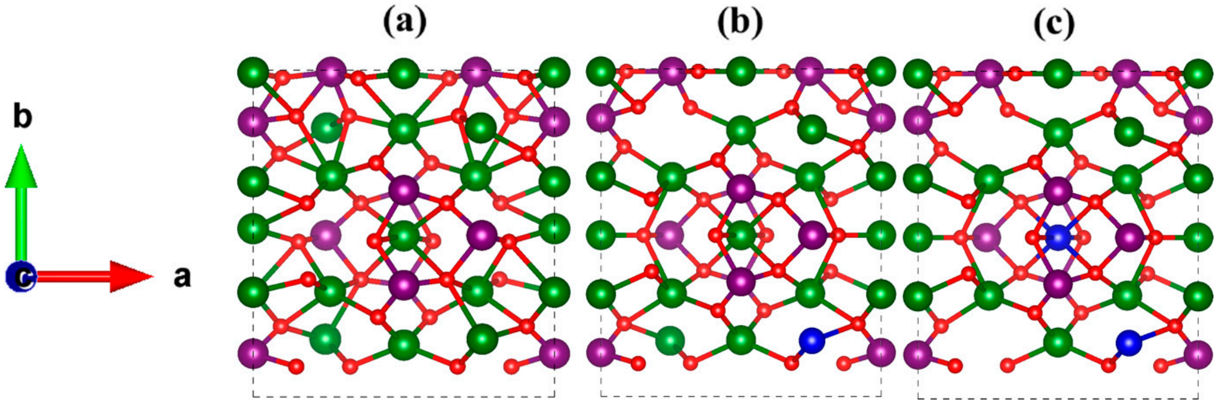

Figure S2. Calculated geometries for α-Ag_2WO_4 undoped (a), doped by one copper atom (b) and doped by two copper atoms (c). The Cu, Ag, W, and O atoms are represented by blue, green, purple, and red colors, respectively.

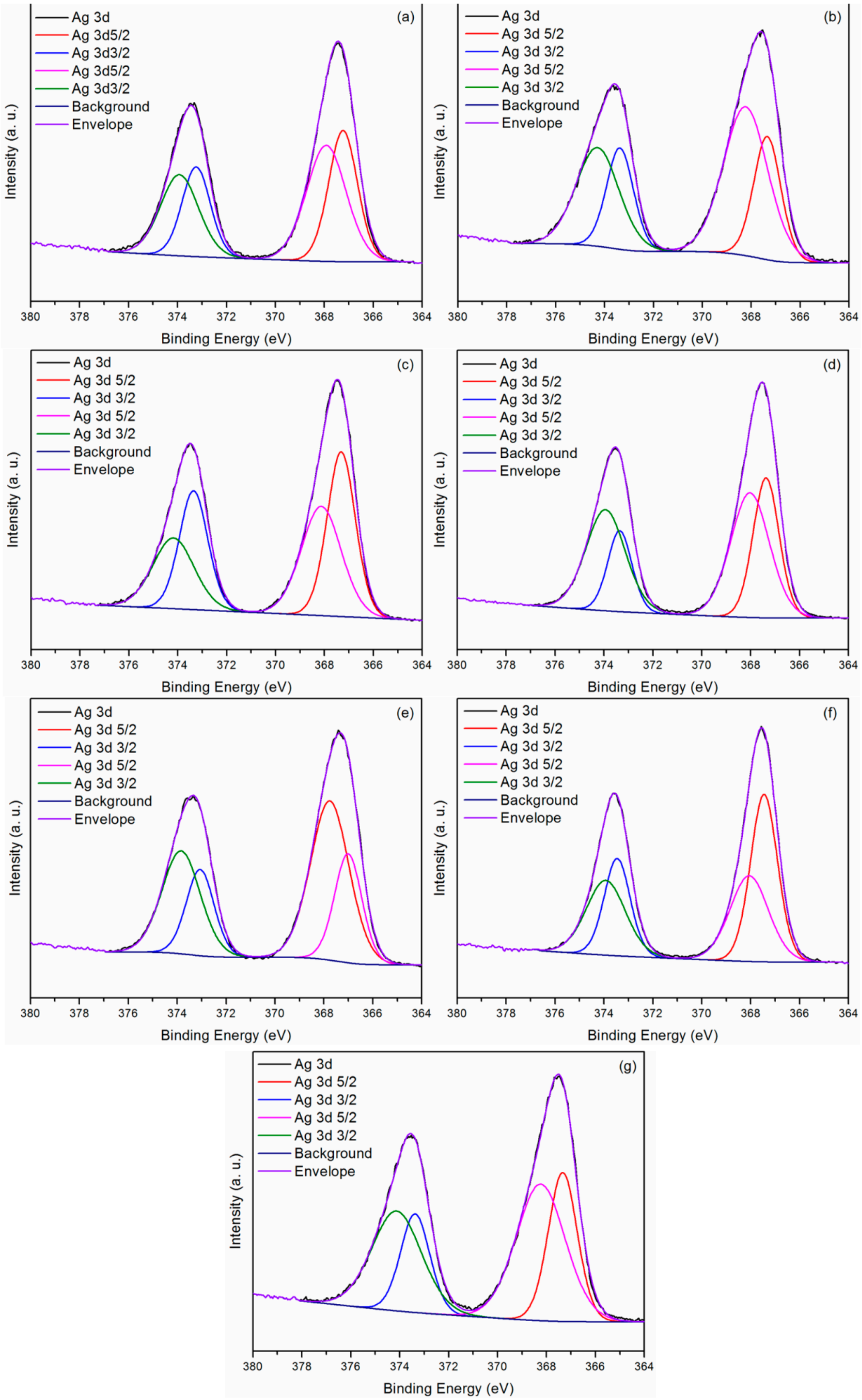

Figure S3. Core level spectrum of (a)-(g) Ag-3*d*; (h)-(n) W-4*f* and (o)-(u) O-1*s*.

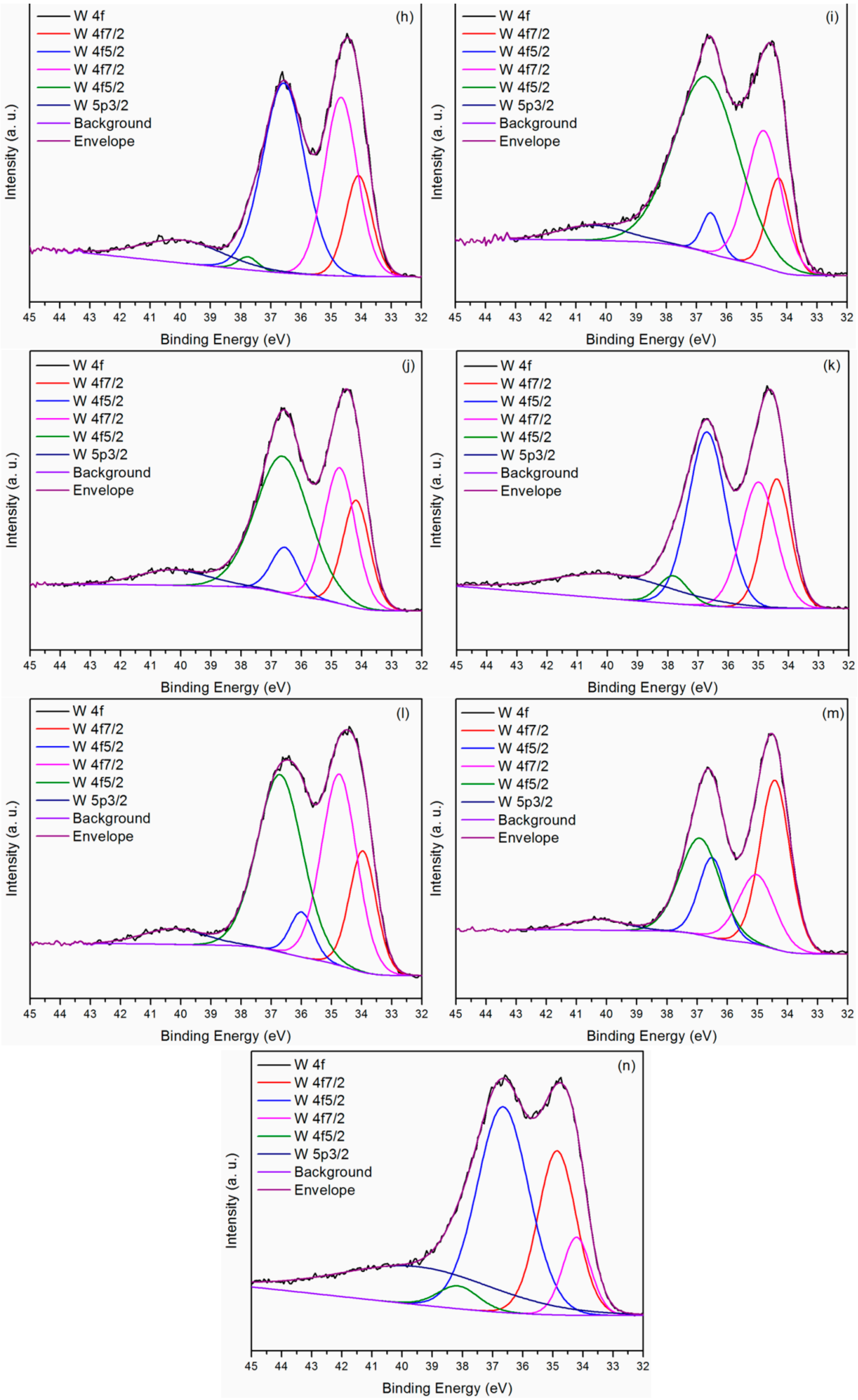

Figure S3 (cont.). Core level spectrum of (a)-(g) Ag-3*d*; (h)-(n) W-4*f* and (o)-(u) O-1*s*.

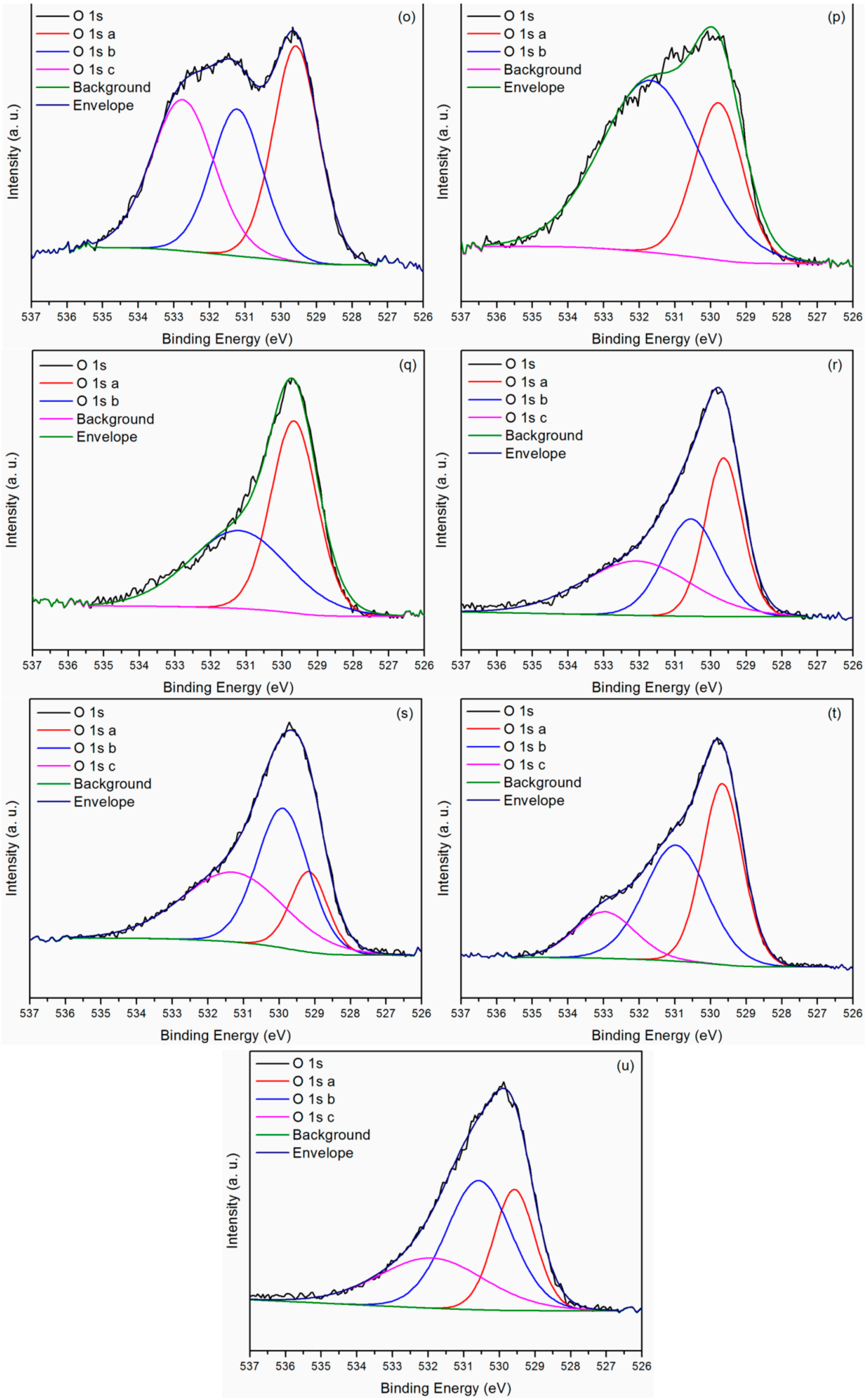

Figure S3 (cont.). Core level spectrum of (a)-(g) Ag-3*d*; (h)-(n) W-4*f* and (o)-(u) O-1*s*.

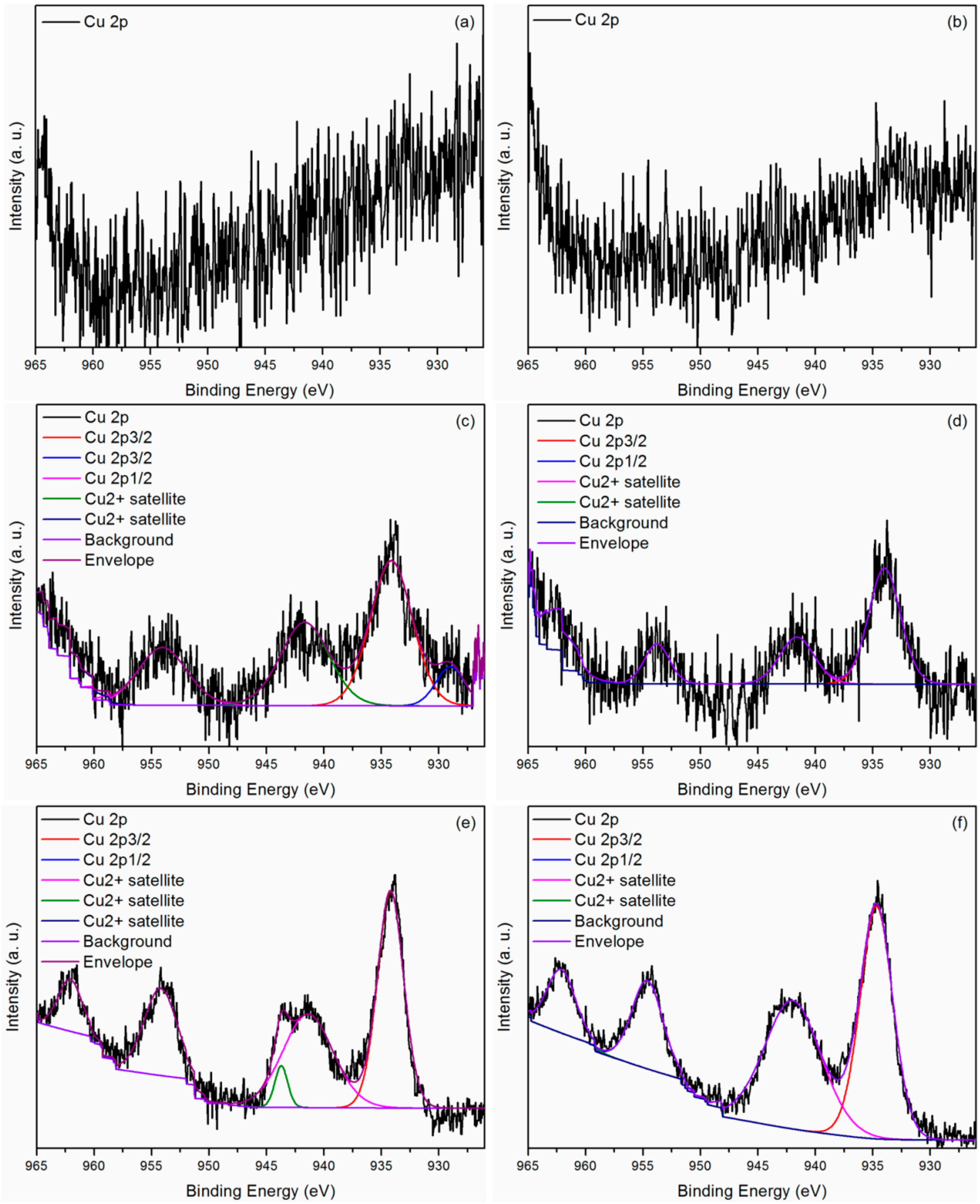

Figure S4. Core level spectrum of Cu-2*p* of the α-$Ag_{2-2x}Cu_xO_4$ solid solutions with (a) $x = 0.005$; (b) $x = 0.01$; (c) $x = 0.02$; (d) $x = 0.04$; (e) $x = 0.08$; and (f) $x = 0.16$.

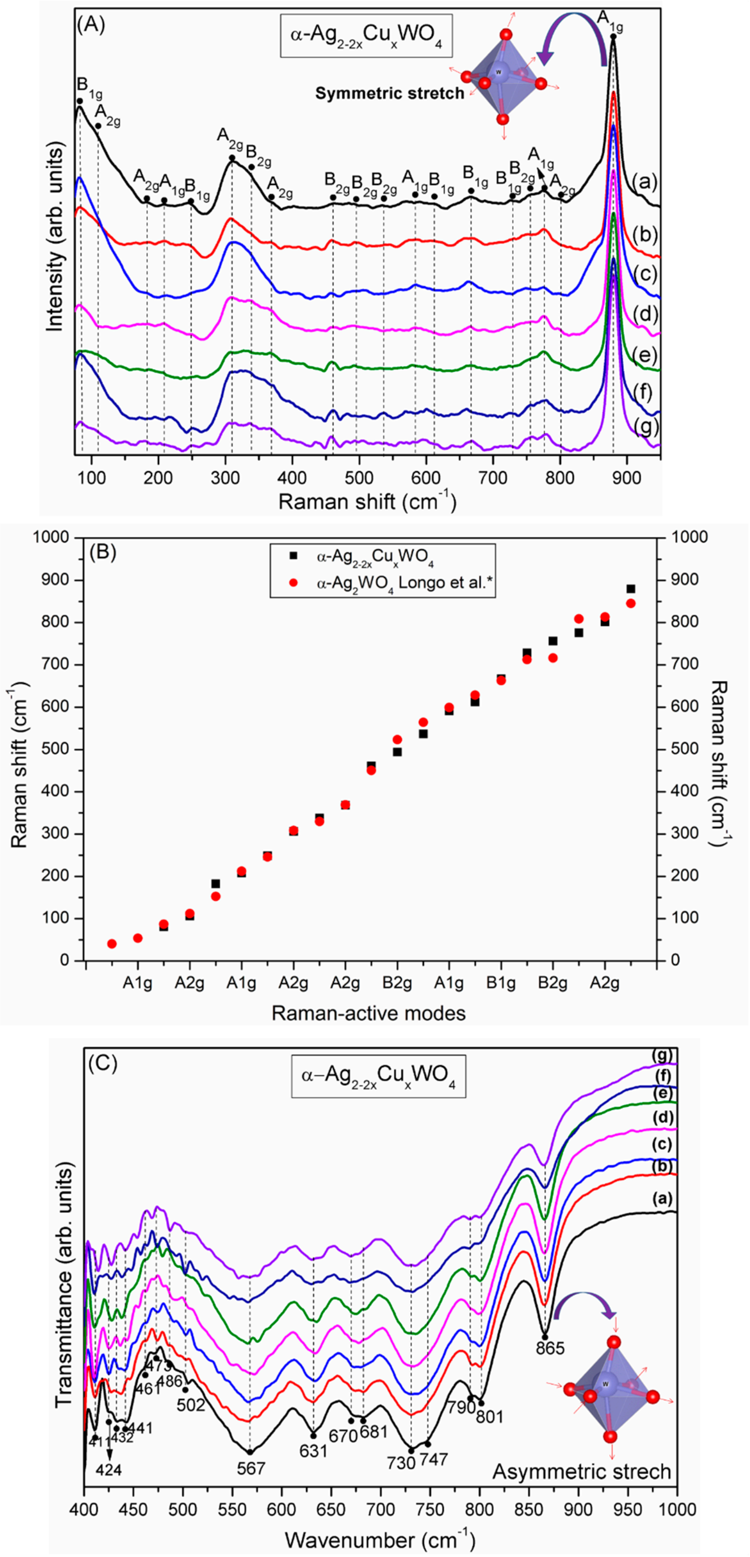

Figure S5. (A and B) Experimental and theoretical Raman-active modes and **(C)** Experimental ATR-FTIR spectra of α-$Ag_{2-2x}Cu_xWO_4$ for x: (a) 0.00, (b) 0.005, (c) 0.01, (d) 0.02, (e) 0.04, (f) 0.08 and (g) 0.16.

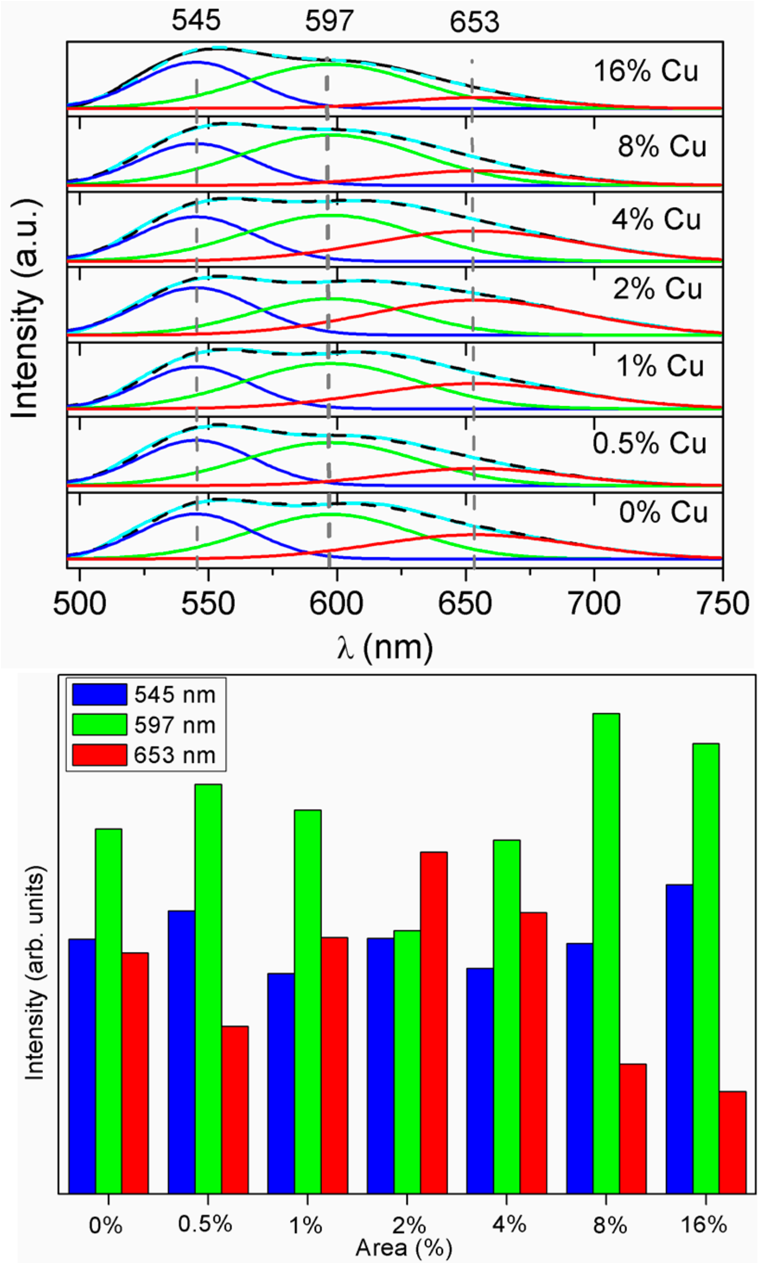

Figure S6. Deconvolution of PL spectra of α-$Ag_{2-2x}Cu_xO_4$ ($0 \leq x \leq 0.16$) solid solutions and area percentage of each color component corresponding to the emission peak.

Tables

Table S1. Lattice parameters, unit cell volume and statistical parameters of quality obtained by Rietveld refinements for the α-$Ag_{2-2x}Cu_xWO_4$ ($0 \leq x \leq 0.16$) solid solutions.

Refined formula α-$Ag_{2-2x}Cu_xWO_4$	Lattice Parameters			Cell volume (Å^3)	R_{Bragg} (%)	χ^2 (%)	R_{wp} (%)	R_p (%)
	a (Å)	b (Å)	c (Å)					
x = 0.00	10.880(0)	12.027(3)	5.901(2)	772.22(7)	2.06	1.60	8.79	6.98
x = 0.005	10.868(1)	12.029(2)	5.902(2)	771.63(2)	1.79	1.74	9.63	7.72
x = 0.01	10.863(8)	12.026(5)	5.899(8)	770.84(5)	1.93	1.67	9.31	7.22
x = 0.02	10.862(4)	12.025(7)	5.902(2)	771.00(5)	2.25	1.98	10.30	8.10
x = 0.04	10.865(1)	12.027(6)	5.897(0)	770.63(6)	2.11	1.66	9.47	7.25
x = 0.08	10.853(6)	12.030(0)	5.899(4)	770.29(1)	2.98	1.45	8.12	6.49
x = 0.16	10.881(0)	12.027(4)	5.890(8)	770.93(9)	2.50	1.43	7.68	6.01
ICSD N°4165	10.89(2)	12.03(2)	5.92(2)	775.56	-	-	-	-

Table S2. Atomic positions of the α-$Ag_{2-2x}Cu_xO_4$ solid solutions with x = 0.00 and 0.005.

α-$Ag_{2-2x}Cu_xO_4$ solid solutions										
Atoms	x = 0.00					x = 0.005				
	x	y	z	Wyckoff Sites	Occupancy (%)	x	y	z	Wyckoff Sites	Occupancy (%)
*W*1	0.2577(5)	-0.0033(1)	0.5170(5)	4 c	100	0.2564(1)	-0.0024(8)	0.5179(5)	4 c	100
*W*2	0.000	0.8441(8)	0.500	2 b	100	0.000	0.8450(1)	0.500	2 b	100
*W*3	0.000	0.1346(8)	0.500	2 b	100	0.000	0.1355(1)	0.500	2 b	100
*Ag*1	0.7518(7)	0.1713(1)	0.9912(3)	4 c	100	0.7557(6)	0.1737(1)	0.9920(0)	4 c	100
*Ag*2	0.2358(7)	0.8178(1)	0.0127(3)	4 c	100	0.2397(6)	0.8202(1)	0.0135(0)	4 c	100
*Ag*3	0.000	0.9874(1)	0.000	2 a	100	0.000	0.9898(1)	0.000	2 a	100
*Ag*4	0.000	0.6533(1)	0.000	2 a	100	0.000	0.6557(1)	0.000	2 a	100
*Ag*5	0.000	0.3150(1)	0.000	2 a	100	0.000	0.3174(1)	0.000	2 a	100
*Ag*6	0.000	0.5094(1)	0.500	2 b	100	0.000	0.5118(1)	0.500	2 b	100
*O*1	0.3698(2)	0.6150(8)	0.1795(1)	4 c	100	0.3686(1)	0.6073(7)	0.1917(1)	4 c	100
*O*2	0.3698(2)	0.3810(8)	0.1725(1)	4 c	100	0.3686(1)	0.3733(7)	0.1847(1)	4 c	100
*O*3	0.4208(2)	0.7380(8)	0.7995(1)	4 c	100	0.4196(1)	0.7303(7)	0.8117(1)	4 c	100
*O*4	0.4268(2)	0.2660(8)	0.7765(1)	4 c	100	0.4256(1)	0.2583(7)	0.7887(1)	4 c	100
*O*5	0.1638(2)	0.4970(8)	0.2665(1)	4 c	100	0.1626(1)	0.4893(7)	0.2787(1)	4 c	100
*O*6	0.4158(2)	0.4990(8)	0.8315(1)	4 c	100	0.4146(1)	0.4913(7)	0.8437(1)	4 c	100
*O*7	0.1908(2)	0.6150(8)	0.8415(1)	4 c	100	0.1896(1)	0.6073(7)	0.8537(1)	4 c	100
*O*8	0.1948(2)	0.3820(8)	0.88450(1)	4 c	100	0.1936(1)	0.3743(7)	0.8967(1)	4 c	100
Cu1	-	-	-	-		-	-	-	-	-
Cu2	-	-	-	-		-	-	-	-	-
Cu3	-	-	-	-		-	-	-	-	-
Cu4	-	-	-	-		-	-	-	-	-
Cu5	-	-	-	-		-	-	-	-	-
Cu6	-	-	-	-		-	-	-	-	-

Table S3. Atomic positions of the α-$Ag_{2-2x}Cu_xO_4$ solid solutions with x = 0.01 and 0.02.

Atoms	α-$Ag_{2-2x}Cu_xO_4$ solid solutions									
	x = 0.01					x = 0.02				
	x	y	z	Wyckoff Sites	Occupancy (%)	x	y	z	Wyckoff Sites	Occupancy (%)
$W1$	0.2564(8)	-0.0031(5)	0.5177(9)	4 c	100	0.2540(0)	0.000	0.5255(0)	4 c	100
$W2$	0.000	0.8443(4)	0.500	2 b	100	0.000	0.8475(0)	0.500	2 b	100
$W3$	0.000	0.1348(4)	0.500	2 b	100	0.000	0.1380(0)	0.500	2 b	100
$Ag1$	0.7559(4)	0.1744(7)	0.9899(3)	4 c	100	0.7509(0)	0.1711(0)	0.9877(0)	4 c	100
$Ag2$	0.2399(4)	0.8209(7)	0.0114(3)	4 c	100	0.2349(0)	0.8176(0)	0.0092(0)	4 c	100
$Ag3$	0.000	0.9905(7)	0.000	2 a	100	0.000	0.9872(0)	0.000	2 a	100
$Ag4$	0.000	0.6564(7)	0.000	2 a	100	0.000	0.6531(0)	0.000	2 a	100
$Ag5$	0.000	0.3181(7)	0.000	2 a	100	0.000	0.3148(0)	0.000	2 a	100
$Ag6$	0.000	0.5125(7)	0.500	2 b	100	0.000	0.5092(0)	0.500	2 b	100
$O1$	0.3701(0)	0.5998(7)	0.1864(4)	4 c	100	0.3680(0)	0.6120(0)	0.1950(0)	4 c	100
$O2$	0.3701(0)	0.3658(7)	0.1794(4)	4 c	100	0.3717(0)	0.3780(0)	0.1880(0)	4 c	100
$O3$	0.4211(0)	0.7228(7)	0.8064(4)	4 c	100	0.4227(0)	0.7350(0)	0.8150(0)	4 c	100
$O4$	0.4271(0)	0.2508(7)	0.7834(4)	4 c	100	0.4287(0)	0.2630(0)	0.7920(0)	4 c	100
$O5$	0.1641(0)	0.4818(7)	0.2734(4)	4 c	100	0.1657(0)	0.4940(0)	0.2820(0)	4 c	100
$O6$	0.4161(0)	0.4838(7)	0.8384(4)	4 c	100	0.4177(0)	0.4960(0)	0.8470(0)	4 c	100
$O7$	0.1911(0)	0.5998(7)	0.8484(4)	4 c	100	0.1927(0)	0.6120(0)	0.8570(0)	4 c	100
$O8$	0.1951(0)	0.3668(7)	0.8914(4)	4 c	100	0.1967(0)	0.3790(0)	0.9000(0)	4 c	100
Cu1	-	-	-	-		-	-	-	-	-
Cu2	-	-	-	-		-	-	-	-	-
Cu3	-	-	-	-		-	-	-	-	-
Cu4	-	-	-	-		-	-	-	-	-
Cu5	-	-	-	-		-	-	-	-	-
Cu6	-	-	-	-		-	-	-	-	-

Table S4. Atomic positions of the α-$Ag_{2-2x}Cu_xO_4$ solid solutions with x = 0.04 and 0.08.

Atoms	α-$Ag_{2-2x}Cu_xO_4$ solid solutions									
	x = 0.04					x = 0.08				
	x	y	z	Wyckoff Sites	Occupancy (%)	x	y	z	Wyckoff Sites	Occupancy (%)
$W1$	0.2577(8)	-0.0019(0)	0.5192(6)	4 c	100	0.2599(0)	-0.0032(9)	0.5274(5)	4 c	100
$W2$	0.000	0.8455(9)	0.500	2 b	100	0.000	0.8442(0)	0.500	2 b	100
$W3$	0.000	0.1360(9)	0.500	2 b	100	0.000	0.1347(0)	0.500	2 b	100
$Ag1$	0.7519(6)	0.1732(0)	0.9917(5)	4 c	100	0.7551(4)	0.1752(5)	0.9922(8)	4 c	84.01
$Ag2$	0.2359(6)	0.8197(0)	0.0132(5)	4 c	100	0.2391(4)	0.8217(5)	0.0137(8)	4 c	100
$Ag3$	0.000	0.9893(0)	0.000	2 a	100	0.000	0.9913(5)	0.000	2 a	100
$Ag4$	0.000	0.6552(0)	0.000	2 a	100	0.000	0.6572(5)	0.000	2 a	100
$Ag5$	0.000	0.3169(0)	0.000	2 a	100	0.000	0.3189(5)	0.000	2 a	100
$Ag6$	0.000	0.5113(0)	0.500	2 b	100	0.000	0.5133(5)	0.500	2 b	100
$O1$	0.3717(7)	0.6016(5)	0.1770(5)	4 c	100	0.3735(3)	0.6031(1)	0.1854(4)	4 c	100
$O2$	0.3717(7)	0.3676(5)	0.1700(5)	4 c	100	0.3735(3)	0.3691(1)	0.1784(4)	4 c	100
$O3$	0.4227(7)	0.7246(5)	0.7970(5)	4 c	100	0.4245(3)	0.7261(1)	0.8054(4)	4 c	100
$O4$	0.4287(7)	0.2526(5)	0.7740(5)	4 c	100	0.4305(3)	0.2541(1)	0.7824(4)	4 c	100
$O5$	0.1657(7)	0.4836(5)	0.2640(5)	4 c	100	0.1675(3)	0.4851(1)	0.2724(4)	4 c	100
$O6$	0.4177(7)	0.4856(5)	0.8290(5)	4 c	100	0.4195(3)	0.4871(1)	0.8374(4)	4 c	100
$O7$	0.1927(7)	0.6016(5)	0.8390(5)	4 c	100	0.1945(3)	0.6031(1)	0.8474(4)	4 c	100
$O8$	0.1967(7)	0.3686(5)	0.8820(5)	4 c	100	0.1985(3)	0.3701(1)	0.8904(4)	4 c	100
Cu1	-	-	-	-	-	0.7509(0)	0.1711(0)	0.9877(0)	4 c	8.0
Cu2	-	-	-	-	-	0.2349(0)	0.8176(0)	0.0092(0)	4 c	0.0
Cu3	-	-	-	-	-	0.000	0.9872(0)	0.000	2 a	0.0
Cu4	-	-	-	-	-	0.000	0.6531(0)	0.000	2 a	0.0
Cu5	-	-	-	-	-	0.000	0.3148(0)	0.000	2 a	0.0
Cu6	-	-	-	-	-	0.000	0.5092(0)	0.500	2 b	0.0

Table S5. Atomic positions of the α-$Ag_{2-2x}Cu_xO_4$ solid solutions with x = 0.16.

Atoms	α-$Ag_{2-2x}Cu_xWO_4$ solid solutions				
	x = 0.16				
	x	y	z	Wyckoff Sites	Occupancy (%)
$W1$	0.2593(2)	-0.0045(2)	0.5202(5)	4 c	100
$W2$	0.000	0.8429(7)	0.500	2 b	100
$W3$	0.000	0.1334(7)	0.500	2 b	100
$Ag1$	0.7542(9)	0.1760(1)	0.9926(1)	4 c	73.82
$Ag2$	0.2382(9)	0.8225(1)	0.0141(1)	4 c	100
$Ag3$	0.000	0.9921(1)	0.000	2 a	94.24
$Ag4$	0.000	0.6580(1)	0.000	2 a	100
$Ag5$	0.000	0.3197(1)	0.000	2 a	100
$Ag6$	0.000	0.5141(1)	0.500	2 b	100
$O1$	0.3704(8)	0.5952(7)	0.1856(0)	4 c	100
$O2$	0.3704(8)	0.3612(7)	0.1786(0)	4 c	100
$O3$	0.4214(8)	0.7182(7)	0.8056(0)	4 c	100
$O4$	0.4274(8)	0.2462(7)	0.7826(0)	4 c	100
$O5$	0.1644(8)	0.4772(7)	0.2726(0)	4 c	100
$O6$	0.4164(8)	0.4792(7)	0.8376(0)	4 c	100
$O7$	0.1914(8)	0.5952(7)	0.8476(0)	4 c	100
$O8$	0.1954(8)	0.3622(7)	0.8906(0)	4 c	100
Cu1	0.7509(0)	0.1711(0)	0.9877(0)	4 c	13.09
Cu2	0.2349(0)	0.8176(0)	0.0092(0)	4 c	0.0
Cu3	0.000	0.9872(0)	0.000	2 a	2.88
Cu4	0.000	0.6531(0)	0.000	2 a	0.0
Cu5	0.000	0.3148(0)	0.000	2 a	0.0
Cu6	0.000	0.5092(0)	0.500	2 b	0.0

Table S6. Values of peaks ratios in At% between C, Ag, W, O, and Cu for typical XPS survey spectra of α-$Ag_{2-2x}Cu_xWO_4$ ($0 \leq x \leq 0.16$) solid solutions.

α-$Ag_{2-2x}Cu_xWO_4$ solid solutions	C-1*s*	Ag-3*d*	W-4*d*	O-1*s*	Cu-2*p*
$x = 0.00$	67.46	7.35	1.23	23.95	-
$x = 0.005$	63.41	7.42	1.89	27.28	-
$x = 0.01$	49.83	13.21	3.87	33.09	-
$x = 0.02$	56.75	9.73	3.56	29.42	0.55
$x = 0.04$	48.93	15.46	3.12	31.95	0.54
$x = 0.08$	58.09	6.88	4.57	30.16	0.30
$x = 0.16$	60.69	5.01	2.56	31.55	0.20

Table S7. FWHM values, intensities, and positions of the Raman peaks of α-$Ag_{2-2x}Cu_xWO_4$ ($0 \leq x \leq 0.16$) solid solutions.

α-$Ag_{2-2x}Cu_xWO_4$ solid solutions	FWHM	Intensities	Positions 2θ (°)
$x = 0.00$	19.148	0.850	879.60
$x = 0.005$	16.598	0.891	879.65
$x = 0.01$	21.844	0.863	878.82
$x = 0.02$	16.687	0.902	879.41
$x = 0.04$	16.911	0.875	879.68
$x = 0.08$	18.171	0.840	879.76
$x = 0.16$	17.791	0.939	880.01
